# Sublimable Spin‐Crossover Complexes: From Spin‐State Switching to Molecular Devices

**DOI:** 10.1002/anie.201911256

**Published:** 2020-10-29

**Authors:** Kuppusamy Senthil Kumar, Mario Ruben

**Affiliations:** ^1^ Institut de Physique et Chimie des Matériaux de Strasbourg (IPCMS) CNRS-Université de Strasbourg 23, rue du Loess, BP 43 67034 Strasbourg cedex 2 France; ^2^ Institute of Nanotechnology Karlsruhe Institute of Technology (KIT) Hermann-von-Helmholtz-Platz 1 76344 Eggenstein-Leopoldshafen Germany; ^3^ Institute of Quantum Materials and -Technology Karlsruhe Institute of Technology (KIT) Hermann-von-Helmholtz-Platz 1 76344 Eggenstein-Leopoldshafen Germany

**Keywords:** magnetic properties, molecular electronics, spin crossover, thin films

## Abstract

Spin‐crossover (SCO) active transition metal complexes are an important class of switchable molecular materials due to their bistable spin‐state switching characteristics at or around room temperature. Vacuum‐sublimable SCO complexes are a subclass of SCO complexes suitable for fabricating ultraclean spin‐switchable films desirable for applications, especially in molecular electronics/spintronics. Consequently, on‐surface SCO of thin‐films of sublimable SCO complexes have been studied employing spectroscopy and microscopy techniques, and results of fundamental and technological importance have been obtained. This Review provides complete coverage of advances made in the field of vacuum‐sublimable SCO complexes: progress made in the design and synthesis of sublimable functional SCO complexes, on‐surface SCO of molecular and multilayer thick films, and various molecular and thin‐film device architectures based on the sublimable SCO complexes.

## Graphical abstract

This review provides a **c**omplete account of the on‐surface spin‐state switching behavior of films of spin‐crossover (SCO) complexes of molecular to multilayer thickness. SCO‐based molecular and thin‐film device architectures fabricated employing vacuum sublimation are also described.

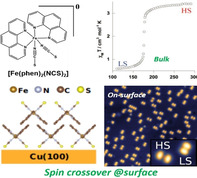

## 1 Introduction

Switchable molecular materials are an important class of molecular materials with potential for applications in molecular electronics, spintronics, information storage, micromechanics, and sensing.[^1^, ^2^, ^3^, ^4^, ^5^, ^6^, ^7^, ^8^, ^9^, ^10^, ^11^] In the emerging area of molecular spintronics, both the electronic charge and spin degrees of freedom are utilized to build device architectures with greater efficiency, both in terms of power and performance.[^9^, ^10^, ^12^, ^13^, ^14^, ^15^, ^16^, ^17^, ^18^, ^19^] Conventional inorganics such as metals and semiconductors are the commonly employed materials to fabricate spintronics architectures.[^9^, ^10^, ^20^] However, the availability of only a few inorganic materials suitable for applications and the difficulties to process them as tailor‐made systems with additional functionalities and tunable quantum states necessitated the search for new materials.^[21]^ Magnetic molecules, comprising paramagnetic transition metal/lanthanide ions in an appropriate ligand field imposed by surrounding ligand(s), are promising molecular spintronic elements due to their quantized energy levels and the tunable nature of their magnetic characteristics.[^16^, ^22^, ^23^, ^24^, ^25^, ^26^, ^27^] Spin‐crossover (SCO) active first‐row transition metal complexes with 3d^*n*^ (*n*=4–7) electronic configuration capable of undergoing reversible interconversion between low‐spin (LS) and high‐spin (HS) states, or vice versa, are an important class of switchable molecular materials. Bi‐ and multistable SCO complexes showing abrupt and stepwise SCO behavior with thermal hysteresis (Δ*T*) are suitable to develop molecular spintronics and memory architectures. The SCO systems are also proposed to be useful developing solvent sensors and micromechanical actuators.[^28^, ^29^, ^30^, ^31^, ^32^, ^33^, ^34^, ^35^, ^36^, ^37^, ^38^, ^39^, ^40^, ^41^, ^42^, ^43^, ^44^]

To realize SCO‐based applications, the spin‐state switching behavior of the complexes must be studied in nanostructured environments, especially in thin‐film form. However, the transition from the bulk to nanometer (nm) regime results in the modulation of intermolecular interactions, thereby SCO in the nanometer regime differs from that in the bulk.^[45]^ The fabrication of SCO complexes as thin films on metallic surfaces creates an interfacial electronic structure (spinterface) due to the electronic coupling between molecules and surface states,[^46^, ^47^, ^48^, ^49^] which alters the SCO behavior of the mono‐ or few‐layer films.[^50^, ^51^] Drop‐casting, spin‐coating, lithographic patterning, Langmuir–Blodgettery (LB), and vacuum sublimation techniques have been utilized to fabricate thin‐film/nanostructured SCO materials.[^52^, ^53^, ^54^, ^55^, ^56^, ^57^, ^58^, ^59^, ^60^, ^61^, ^62^, ^63^] Among the techniques, direct vacuum sublimation of SCO complexes onto suitable surfaces emerged as a preferred method to obtain ultraclean and high‐quality spin‐switchable thin films.[^64^, ^65^, ^66^, ^67^, ^68^, ^69^, ^70^, ^71^, ^72^, ^73^, ^74^, ^75^] Charge‐neutral SCO complexes with a propensity to undergo sublimation are suitable candidates to prepare mono/multilayer thin spin‐switchable films—results of fundamental and applied scientific importance, such as the elucidation of the electron‐induced SCO^[76]^ and memristance behavior at the single‐molecule scale^[77]^ have been reported. Despite the enormous application potential envisioned and significant advances made in terms of studying SCO in thin sublimed films, a definitive account covering SCO of sublimable SCO complexes in the bulk and the thin‐film states, and device architectures based on SCO films is yet to be presented in the form of a review. We aim to bridge this gap by presenting an account detailing development made in the field of sublimable SCO materials. The emphasis is placed on the elucidation of physicochemical aspects governing the on‐surface spin‐state switching and device architectures based on thin vacuum‐sublimed SCO films.

For a conceptual introduction of the SCO phenomenon, the reader is advised to consult the brief and concise overview presented by Gütlich and Garcia^[78]^ and other informative reviews and books.[^31^, ^42^, ^79^] To aid the reader, the most frequently used concepts are defined as follows: The temperature at which equal proportions of LS and HS states coexist is denoted as *T*_1/2_. The term “light‐induced SCO” ^[80]^ is a general term utilized to refer to light‐induced excited spin‐state trapping (LIESST) mediated LS→HS switching below 10 K.[^40^, ^41^] The metastable HS state produced via the LIESST effect is stable only up to a temperature denoted as *T*_(LIESST)_, above which the HS system relaxes back to the corresponding LS state. The analogous term “soft X‐ray induced excited spin‐state trapping” (SOXIESST) is a process in which soft X‐ray irradiation mediates LS→HS switching at low temperatures.[^81^, ^82^] The SOXIESST effect is of importance when SCO of mono/multilayer films of SCO complexes is probed with X‐ray absorption spectroscopy (XAS).

## 2 Spin‐State Switching in the Bulk

**Scheme 1:**
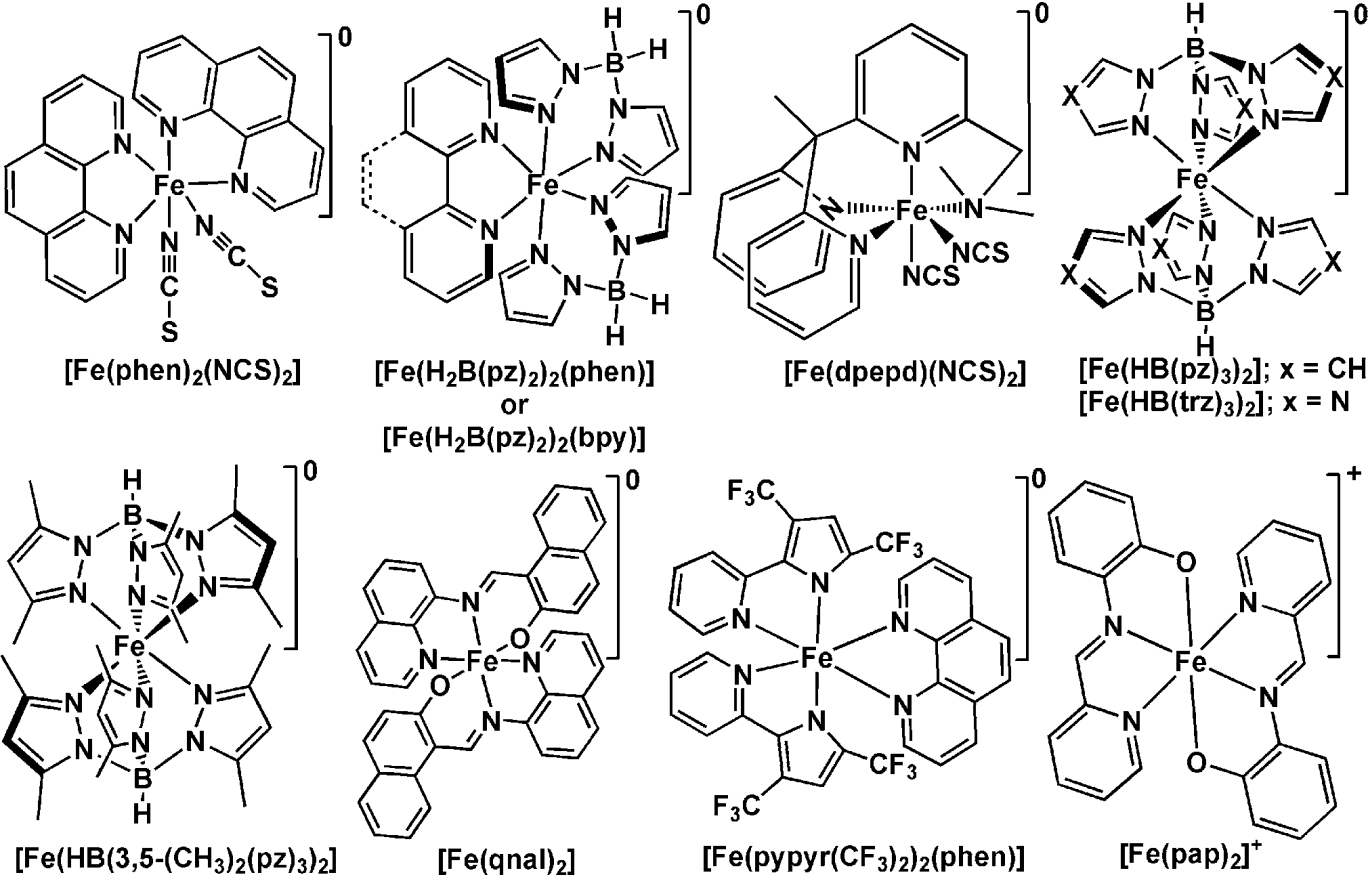
Molecular structures of the sublimable SCO complexes.

Up to now, only a handful of charge‐neutral Fe^II^‐SCO complexes (see Scheme 1 and Table 1) have been reported that undergo sublimation. The Fe^III^ complex [Fe(pap)_2_]ClO_4_⋅H_2_O is the only charged complex so far reported that undergoes sublimation.^[83a]^

**Table 1:** Bulk *T*_1/2_, Δ*T*, *T*_LIESST_, sublimation temperature (*T*_sub_), and sublimation pressure (*P*_sub_) of the sublimable SCO complexes.

Molecule	*T*_1/2/_Δ*T* (K)^[a]^	*T*_LIESST_ (K)	*T*_sub_ (K)/*P*_sub_ (mbar)	Ref
[Fe(phen)_2_(NCS)_2_]	176/≈1	62	453/10^−8^	[67]
[Fe(H_2_B(pz)_2_)_2_(phen)]	163.7/≈4	44	435/10^−2^	[86]
[Fe(H_2_B(pz)_2_)_2_(bpy)]	160	52	433/10^−2^	[86]
[Fe(dpepd)(NCS)_2_]	≈251		510/5×10^−9^	[90]
[Fe(HB(pz)_3_)_2_]	393		463/≈10^−5^	[91, 92]
[Fe(HB(trz)_3_)_2_]	333/≈2		523/≈2×10^−7^	[93]
[Fe(HB(3,5‐(CH_3_)_2_(pz)_3_)_2_]	189/31		393‐413/10^−8^	[94]
[Fe(qnal)_2_]⋅*x* CH_2_Cl_2_	225 (*x=*1) and 260 (*x=*0)	≈60	490/10^−7^	[72]
[Fe(pypyr(CF_3_)_2_)_2_(phen)]	390	not active	433/5×10^−9^	[95]
[Fe(pap)_2_]ClO_4_⋅H_2_O	172.5/15	≈70	353‐373/≈1×10^−9^	[83, 96]

[a] From SQUID measurements.

Among the so far reported sublimable SCO complexes, [Fe(phen)_2_)(NCS)_2_],[^1^, ^84^, ^85^] [Fe(H_2_B(pz)_2_)_2_(phen)], [Fe(H_2_B(pz)_2_)_2_(bpy)], and Fe[HB(trz)_3_]_2_ (Scheme 1)^[86]^ are well‐studied on surface, as detailed in Section 3.

**Figure 1:**
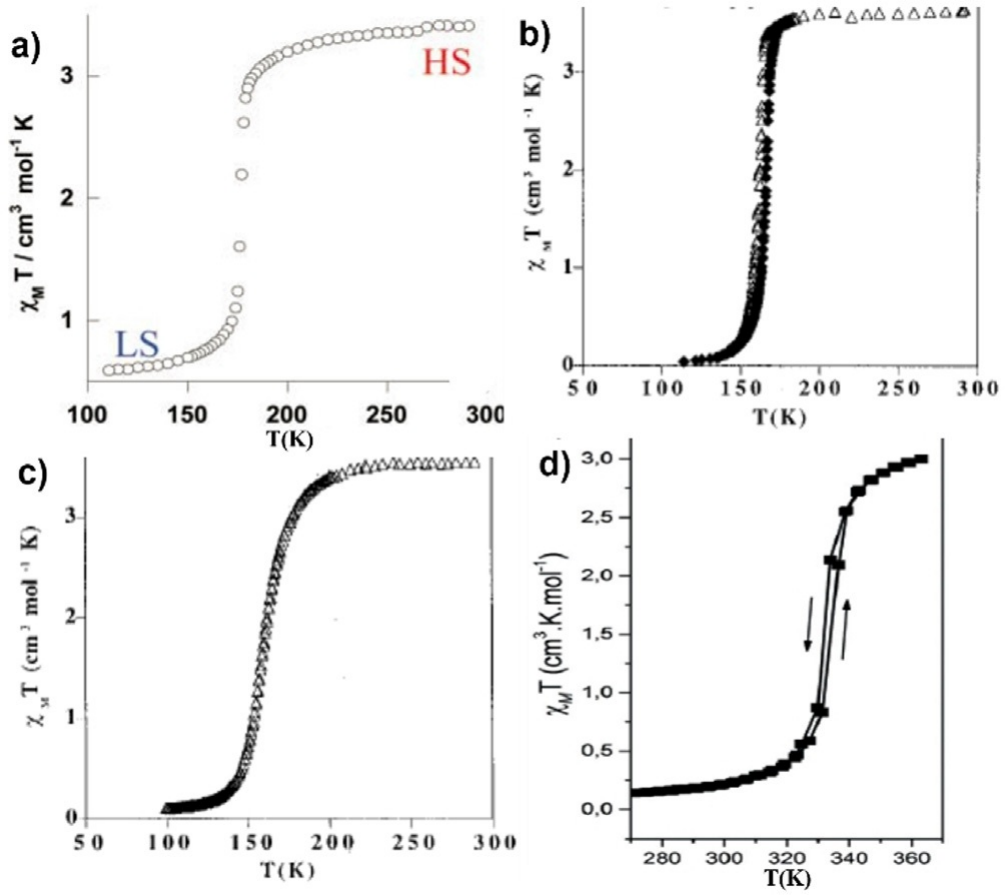
The bulk state SCO behavior (*χ*_M_ *T* versus *T* plots) of the four most studied sublimable SCO complexes on surface. a) [Fe(phen)_2_(NCS)_2_], b) [Fe(H_2_B(pz)_2_)_2_(phen)], c) [Fe(H_2_B(pz)_2_)_2_(bpy)], and d)  Fe[HB(trz)_3_]_2_. The images in (a), (b,c), and (d) are reproduced with permission from ref. [44] (copyright (2005) Royal Society of Chemistry), ref. [86] (copyright (1997) American Chemical Society) and ref. [93] (copyright (2017) Royal Society of Chemistry), respectively.

The prototypical complex, [Fe(phen)_2_(NCS)_2_], undergoes abrupt HS→LS switching upon cooling with *T*_1/2_=176 K as depicted in Figure 1 a. Low‐temperature irradiation of the complex with visible or ultraviolet light caused LIESST‐mediated LS→HS switching (*T*_(LIESST)_=62 K). A more detailed analysis of thermal and light‐induced SCO of [Fe(phen)_2_(NCS)_2_] is reported elsewhere.[^87^, ^88^] The complex is also SOXIESST active upon soft X‐ray irradiation at the iron L_2,3_ edges at 45 K. Prolonged irradiation of the complex produced a thermally irreversible low‐spin (LS′) state via soft X‐ray photochemistry (SOXPC), indicating the sensitive nature of the complex to X‐ray flux.^[81]^ Upon size reduction, the rhombohedric nanoplatelets of the complex showed more abrupt and cooperative thermal SCO, with *T*_1/2_=180 K and Δ*T*=8 K, relative to the bulk complex.^[89]^ An analogous complex, [Fe(dpepd)(NCS)_2_], showed a gradual, complete, and one‐step SCO with *T*_1/2_≈251 K (Figure S1).^[90]^

Complexes [Fe(H_2_B(pz)_2_)_2_(phen)] and [Fe(H_2_B(pz)_2_)_2_(bpy)] showed HS→LS switching upon cooling with *T*_1/2_≈160 K; Δ*T*=4 K is observed for [Fe(H_2_B(pz)_2_)_2_(phen)] (Figure 1 b,c and Table 1). The [Fe(H_2_B(pz)_2_)_2_(phen)] and [Fe(H_2_B(pz)_2_)_2_(bpy)] complexes are also LIESST active with *T*_(LIESST)_=44 K and 52 K, respectively.[^86^, ^97^] The bulk microcrystalline Fe[HB(pz)_3_]_2_, obtained from vacuum sublimation of the as‐synthesized powder, showed an incomplete, gradual, and hysteretic SCO in the first heating–cooling cycle, and subsequent cycles showed no hysteresis (Figure S2 a).^[91]^ Conversely, the CH_3_‐substituted complex [Fe(HB(3,5‐(CH_3_)_2_(pz)_3_)_2_] showed an abrupt, complete, and hysteretic SCO with *T*_1/2_≈189 K and Δ*T*=31 K (Figure S2 b).^[94]^ Slightly different SCO parameters, *T*_1/2_≈187 K and Δ*T*=25 K, were reported for the same complex in another study.^[66]^ The discrepancy may be due to the differing crystallite size of the complex prepared and studied by two different groups.

**Figure 2:**
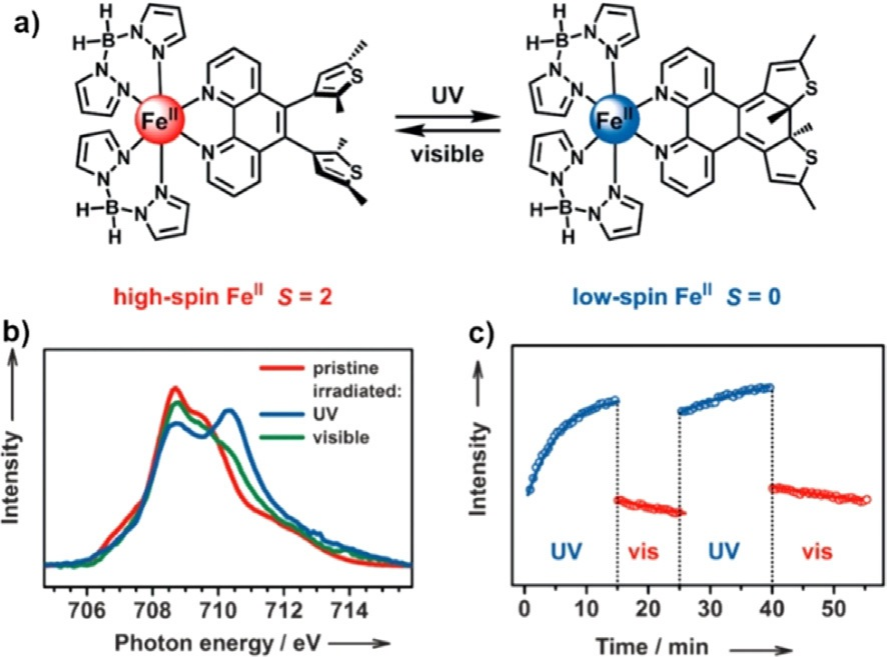
a) Reversible photo‐induced ring closure and opening of the diarylethene‐tethered sublimable SCO complex [Fe(H_2_B(pz)_2_)_2_(L^8^)]⋅H_2_O in the solid state; b) XAS of the open‐ring (HS) form at the Fe‐L_3_ edge at RT (red), after UV light irradiation (blue), and after visible‐light irradiation (green); c) multiple photo‐switching of the complex evidenced by the intensity variation of the Fe‐L_3_ peak at 710.2 eV corresponding to the LS state of the complex. Reproduced with permission from reference 107. Copyright (2013) American Chemical Society.

Remarkably, [Fe(HB(trz)_3_)_2_] showed an abrupt SCO above RT with *T*_1/2_=333 K and Δ*T*=2 K (Figure 2 d).^[93]^ Complex [Fe(qnal)_2_] showed abrupt and lattice solvent dependent SCO (Figure S3) with *T*_1/2_=260 K ([Fe(qnal)_2_]) and 225 K ([Fe(qnal)_2_]CH_2_Cl_2_).^[72]^ An incomplete, gradual, and above RT SCO (*T*_1/2_=390 K; Figure S4) was observed for [Fe(pypyr(CF_3_)_2_)_2_(phen)].^[95]^ Complex [Fe(pap)_2_]ClO_4_⋅H_2_O showed abrupt and hysteretic SCO (Figure S5) with *T*_1/2_≈173 K (Δ*T*=15 K); the complex is also LIESST active with *T*_(LIESST)_=≈70 K.^[96]^

The chemistry of the [Fe(H_2_B(pz)_2_)_2_(phen)] and [Fe(H_2_B(pz)_2_)_2_(bpy)] family of complexes has been exploited to an extent by tailoring the systems with functional substituents at various positions of the 1,10‐phenanthroline or 2,2′‐bipyridyl rings.[^98^, ^99^, ^100^, ^101^, ^102^] The bulk SCO of functional SCO complexes studied on a surface is described as follows: The SCO behavior of four analogous [Fe(H_2_B(pz)_2_)_2_(L^*n*^)] complexes (*n*=1–4; L^1^=4‐methyl‐1,10‐phenanthroline; L^2^=5‐chloro‐1,10‐phenanthroline; L^3^=4,7‐dichloro‐1,10‐phenanthroline; L^4^=4,7‐dimethyl‐1,10‐phenanthroline) were studied in the bulk and thin‐film states. The complexes with single chloro/methyl substituents, [Fe(H_2_B(pz)_2_)_2_(L^*n*^)] (*n*=1–2), showed thermal and light‐induced SCO in the bulk, whereas the bisubstituted complexes, [Fe(H_2_B(pz)_2_)_2_(L^*n*^)] (*n*=3–4), were trapped in the HS state. However, both the mono‐ and bisubstituted complexes showed thermal and light‐induced SCO in the thin‐film state. Intermolecular π–π interaction mediated dimer formation between neighboring ligands blocked the SCO of [Fe(H_2_B(pz)_2_)_2_(L^*n*^)] (*n*=3–4) in the bulk.^[64]^ A similar complex, [Fe(H_2_B(pz)_2_)_2_(L^5^)] (L^5^=3,4,7,8‐tetramethyl‐1,10‐phenanthroline) featuring four methyl substituents at the phenanthroline ring, was prepared to minimize the van der Waals interaction between the complex and substrate. In the bulk state, [Fe(H_2_B(pz)_2_)_2_(L^5^)] showed a gradual and incomplete SCO with *T*_1/2_=141 K.^[103]^ The 5‐amino‐1,10‐phenanthroline (L^6^)‐based complex [Fe(H_2_B(pz)_2_)_2_(L^6^)] showed thermal SCO with *T*_1/2_=154 K in the bulk state.^[104]^ A pseudo‐amphiphile‐like SCO complex, [Fe(H_2_B(pz)_2_)_2_(L^7^)] (L^7^=dodecyl[2,2′‐bipyridine]‐5‐carboxylate, is the only reported example belonging to the [Fe(H_2_B(pz)_2_)_2_(bpy)] family to have been sublimed on surface. The complex showed complete SCO in the bulk state with *T*_1/2_=210 K.^[105]^

A photochromic diarylethane‐based charge‐neutral Fe^II^ complex, [Fe(H_2_B(pz)_2_)_2_(L^8^)]⋅H_2_O (L^8^=5,6‐bis(2,5‐dimethyl‐3‐thienyl)‐1,10‐phenanthroline), showed reversible ligand‐driven light‐induced spin change (LD‐LISC). The reversible photocyclization propensity of open‐ring (O)‐L^8^ was used to tune the spin state of the corresponding Fe^II^ complexes.[^106^, ^107^] Open‐ring complex O‐[Fe(H_2_B(pz)_2_)_2_(L^8^)]⋅H_2_O (Figure 2 a) is HS at RT and showed thermal SCO upon cooling with *T*_1/2_=140 K; the complex is also LIESST active with *T*_(LIESST)_=52 K.^[106]^

Room‐temperature irradiation of the HS open‐ring complex with UV light (*λ*=254 nm) caused photocyclization of the ligand (Figure 2 a), which in turn led to LD‐LISC mediated HS→LS switching in solution with 40 % efficiency.^[107]^ The reverse process is accomplished by visible light irradiation (*λ*>400 nm) of the closed ring complex. Photo‐irradiation of the open‐ring complex in the solid state resulted in LD‐LISC mediated spin‐state conversion with 32 % efficiency.^[108]^ Mechanistically, the lack of electronic interaction between the phenanthroline and the appended photochromic units favored the HS state of the open‐ring complexes, whereas photo‐induced ring closure increased the conjugation and consequently the π‐accepting character of the closed‐ring ligand, which favored the LS‐state of the closed‐ring complexes.

## 3 On‐Surface SCO

The spin‐state switching behavior of sublimable SCO complexes detailed in Section 2 has been probed on various surfaces at sub‐monolayer to multilayer coverage, as detailed below.

### 3.1 [Fe(phen)_2_(NCS)_2_]

**Figure 3:**
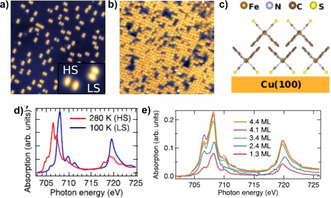
STM images of [Fe(phen)(NCS)_2_] on Cu(100) at a) 0.1 ML coverage (the inset shows the HS and LS states of [Fe(phen)(NCS)_2_] trapped on the surface) and b) at 1.8 ML coverage. c) Molecular organization of [Fe(phen)(NCS)_2_] in a bilayer film. XAS of d) bulk [Fe(phen)(NCS)_2_] at 280 K and 100 K, and e) thin‐film state at 100 K. Reproduced with permission from ref. [67]. Copyright (2017) American Institute of Physics.

Complex [Fe(phen)_2_(NCS)_2_], showing *T*_1/2_=176 K in the bulk state, is the first example of a vacuum‐sublimable SCO complex studied on a surface. A 280 nm film of the complex on a silicon (Si) substrate underwent gradual SCO with *T*_1/2_≈175 K, which is comparable to the bulk *T*_1/2_≈176 K. However, the 1 K thermal hysteresis observed for the bulk sample has vanished in the thin film.^[69]^ The occurrence of SCO at the single‐molecule level depends on the nature of molecule–surface interactions. At 4 K, a spin‐state coexistence was observed for a sub‐monolayer film of [Fe(phen)_2_(NCS)_2_] on Cu(100). The appearance of the complexes as two‐lobed structures, in the STM image, corresponding to the upward‐pointing phenanthroline ligands, indicates chemisorption between the NCS groups and Cu(100) surface (Figure 3 c).^[67]^ The increased (HS) and decreased (LS) spatial separation between the lobes facilitated identification of the spin‐state of [Fe(phen)_2_(NCS)_2_] at sub‐monolayer coverage (Figure 3 a).

The insertion of a passive copper nitride (CuN) layer between the Cu(100) surface and [Fe(phen)_2_(NCS)_2_] prevented the chemisorption between the complex and the Cu(100) surface, facilitating a reversible STM‐tip‐mediated SCO, as detailed in Section 4.1.^[77]^ A computational study^[109]^ validates the above experimental findings, demonstrating the role of molecule–surface interactions—chemisorption—in determining the spin‐state of surface‐deposited [Fe(phen)_2_(NCS)_2_] at sub‐monolayer coverage.

At 4 K, the spin‐state co‐existence is reduced for 1.3–4.4 monolayer (ML) films of [Fe(phen)_2_(NCS)_2_] on Cu(100). An increase in the HS fraction with increasing coverage is observed, as inferred from the XAS spectra in Figure 3 e, which is contrary to the LS‐state of the complex, at 4 K, in the bulk‐state (Figures 1 a and 3 d). Due to the different orientation of [Fe(phen)(NCS)_2_] in the second layer, relative to its orientation in the first layer on Cu(100) (Figure 3 b,c), the topography of the complexes residing in the second layer is different, hindering the unambiguous assignment of the spin‐state of the complex based on topography. To elucidate the spin‐state of [Fe(phen)(NCS)_2_] complexes residing in the second layer, differential conductance spectra (d*I/*d*V*) profiles of the reference HS complex in the first monolayer and complexes in the second layer were obtained by performing scanning tunneling spectroscopy (STS). The similarity of the d*I/*d*V* profiles observed for the reference HS molecule and the molecules in the second layer unambiguously indicated the dominant HS character of the molecules in the second layer.

### 3.2 The [Fe(H_2_B(pz)_2_)_2_(phen)] and [Fe(H_2_B(pz)_2_)_2_(bpy)] Family of Complexes

On‐surface SCO of [Fe(H_2_B(pz)_2_)_2_(phen)] and [Fe(H_2_B(pz)_2_)_2_(bpy)], showing *T*_1/2_=164 K and 160 K, respectively, in the bulk, has been studied in detail by subliming the complexes on a range of substrates. Films of the complexes, several hundred nanometers thick, on glass and Kapton tape showed gradual SCO relative to their bulk counterparts. The *T*_1/2_ values of 151 K and 153 K observed for [Fe(H_2_B(pz)_2_)_2_(phen)] and [Fe(H_2_B(pz)_2_)_2_(bpy)], respectively, in the thin‐film state are lower than the bulk values. Remarkably, the thin films of [Fe(H_2_B(pz)_2_)_2_(phen)] and [Fe(H_2_B(pz)_2_)_2_(bpy)] on Kapton tape showed Δ*T*=6 K. Note: only [Fe(H_2_B(pz)_2_)_2_(phen)] in bulk showed hysteretic SCO with Δ*T*=4 K. The complexes are also LIESST active in the thin‐film state and showed *T*_(LIESST)_ values (see Table 1) comparable to those of the bulk samples.^[71]^

**Figure 4:**
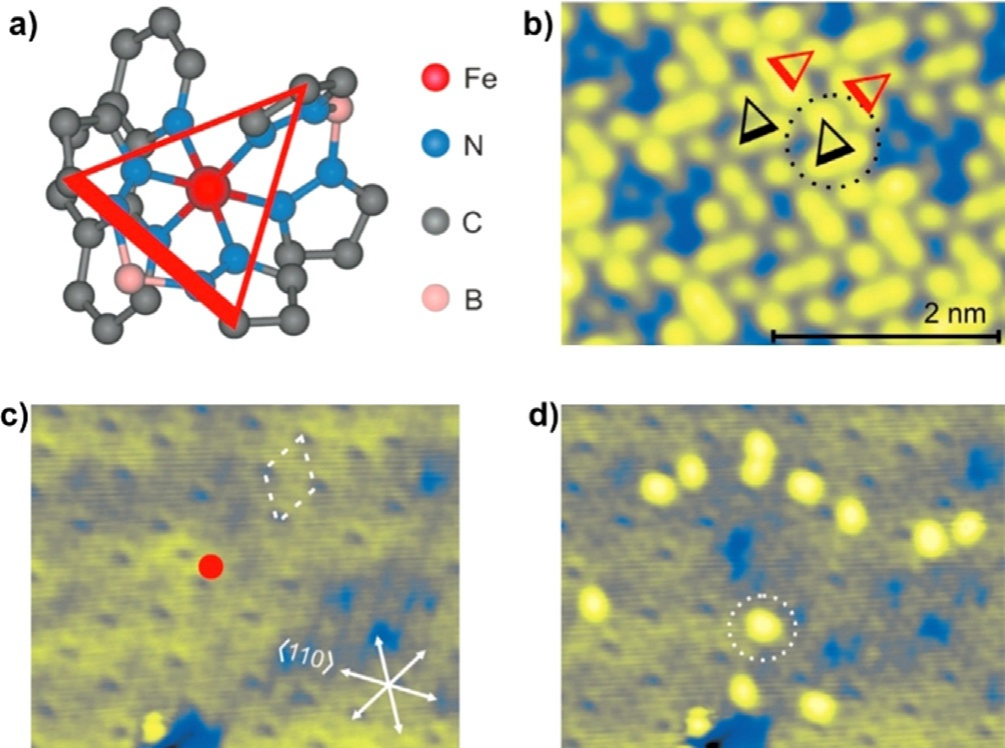
ELIESST in [Fe(H_2_B(pz)_2_)_2_(phen)]. a) View of [Fe(H_2_B(pz)_2_)_2_(phen)] along the pseudo‐trigonal axis. A triangle connecting three pyrazole units of the two different [H_2_B(pz)_2_] ligands is used to represent the molecular orientation of [Fe(H_2_B(pz)_2_)_2_(phen)] on the surface; b) organization of [Fe(H_2_B(pz)_2_)_2_(phen)] on the second layer (yellow); c) large‐area view of the 1.6 ML of [Fe(H_2_B(pz)_2_)_2_(phen)], the red dot indicates the place where the voltage pulse was applied; d) image showing LS→HS switching of the molecules away from the place where the voltage pulse was applied. Note, the HS [Fe(H_2_B(pz)_2_)_2_(phen)] appears bright in the images.

To understand the role of surface states affecting SCO and coverage‐dependent switching, a molecular level study was performed by depositing [Fe(H_2_B(pz)_2_)_2_(phen)] on Au(111) by keeping the substrate at RT. At sub‐monolayer and monolayer coverages, surface‐induced fragmentation of [Fe(H_2_B(pz)_2_)_2_(phen)] into [Fe(H_2_B(pz)_2_)_2_] and phen was observed. However, the complex remained intact in the second layer and showed a gradual and incomplete SCO. The remaining LS complexes in the second layer underwent SOXIESST‐mediated LS→HS switching below 90 K. Deposition of [Fe(H_2_B(pz)_2_)_2_(phen)] onto a Au(111) substrate held in the temperature range of 373 K–423 K reduced fragmentation and facilitated the arrangement of intact [Fe(H_2_B(pz)_2_)_2_(phen)] molecules as an ordered phase at sub‐monolayer coverage. On Au(111), a threefold‐symmetric molecular pattern (Figure 4 a) is observed for [Fe(H_2_B(pz)_2_)_2_(phen)] with the phen lying almost flat on the surface. The on‐surface flat organization of the phen ligand induced fragmentation as it is not compatible with the 3D structure of [Fe(H_2_B(pz)_2_)_2_(phen)].^[110]^

Electron‐induced excited spin‐state trapping (ELIESST) at low temperatures was demonstrated for [Fe(H_2_B(pz)_2_)_2_(phen)] in an STM‐tip/[Fe(H_2_B(pz)_2_)_2_(phen)] (2 layers)/Au(111) junction. The complexes in the second layer showed LS→HS switching upon application of a voltage pulse (*V=*3 V) at a remote place (Figure 4 c). The reverse HS→LS switching was induced by applying a voltage pulse of *V=*1.8 V, by placing the STM tip right above a freshly switched HS molecule. Transportation of STM‐injected hot electrons by surface states of Au(111) across nm distances caused remote SCO of [Fe(H_2_B(pz)_2_)_2_(phen)].^[76]^

To prevent surface‐induced fragmentation, a functional variant of [Fe(H_2_B(pz)_2_)_2_(phen)], [Fe(H_2_B(pz)_2_)_2_(L^5^)], featuring methyl groups at the 3‐, 4‐, 7‐, and 8‐positions of the 1,10‐phenanthroline ring was designed. A thick film of the complex on glass showed thermal (*T*_1/2_=148 K) and light‐induced (*T*_(LIESST)_=54 K) SCO. The thermal SCO of the glass‐surface‐bound film is more gradual and proceeded at a higher temperature relative to the bulk complex. Contrary to the expectations, [Fe(H_2_B(pz)_2_)_2_(phenMe_4_)] underwent fragmentation upon deposition on Au(111) as a sub‐monolayer (0.6 ML) film. However, [Fe(H_2_B(pz)_2_)_2_(phenMe_4_)] remained intact on the semimetal Bi(111) surface at 0.3 ML coverage. A co‐existence of spin states, with 49 % of the complexes undergoing HS→LS switching upon cooling, was observed for the Bi(111)‐bound films, and the LS complexes showed LIESST‐mediated SCO at 17 K. The parent [Fe(H_2_B(pz)_2_)_2_(phen)] showed similar thermal and light‐induced SCO on Bi(111) with a fraction of the complexes permanently trapped in the HS‐state.^[103]^

**Figure 5:**
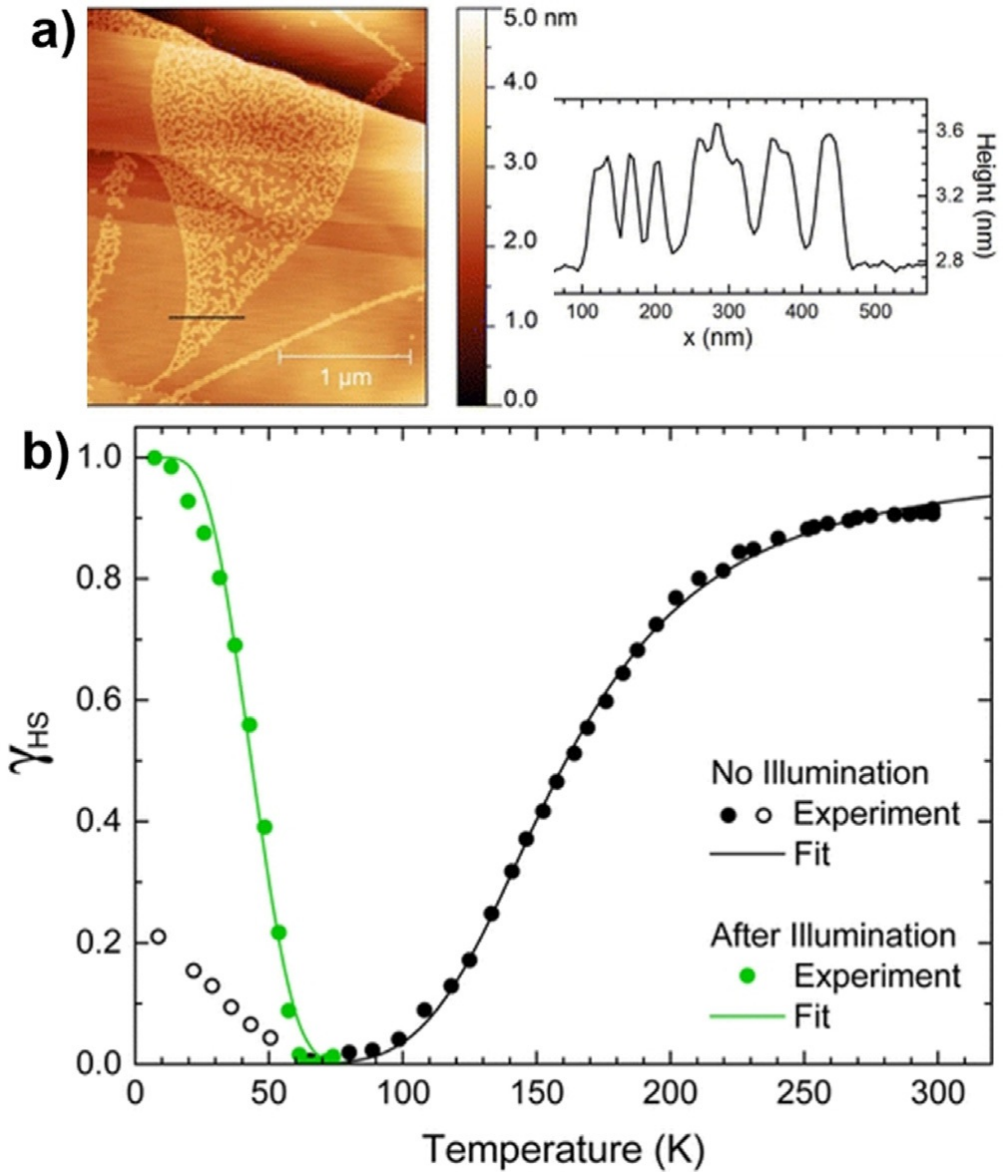
a) STM image and the line profile showing the heights of single [Fe(H_2_B(pz)_2_)_2_(Phen)] molecules on HOPG and b) plot of HS fraction (*γ*_HS_) versus *T* showing complete thermal and LIESST‐mediated spin‐state switching of the [Fe(H_2_B(pz)_2_)_2_(Phen)] on an HOPG surface at sub‐monolayer coverage. Reproduced with permission from ref. [75]. Copyright (2015) American Chemical Society.

To avoid fragmentation, [Fe(H_2_B(pz)_2_)_2_(phen)] was deposited on a passive yet electrically conductive HOPG substrate. STM analysis of the sub‐monolayer film of [Fe(H_2_B(pz)_2_)_2_(phen)] revealed the intact molecular structure of the complex in direct contact with HOPG, as shown in Figure 5 a. Remarkably, the films underwent complete thermal and LIESST‐mediated SCO at sub‐monolayer coverage (Figure 5 b).^[75]^

The realization of RT SCO in surface‐bound thin films is a criterion for the development of SCO‐based applications. In the quest to achieve this, LD‐LISC mediated spin‐state modulation in a 5 nm film of [Fe(H_2_B(pz)_2_)_2_(L^8^)] deposited on a Au(111) surface was studied. UV light (*λ*=285 nm) irradiation of the film at RT induced photocyclization of the open‐ring HS isomer to the closed‐ring LS isomer, thereby effecting HS→LS switching in about 5 % of the molecules. The HS molecules in the as‐sublimed film also showed thermal SCO; a more gradual and incomplete thermal SCO is observed, relative to the bulk phase, upon cooling. The LIESST‐mediated LS→HS switching is observed at low temperatures for the 5 nm film.^[111]^

**Figure 6:**
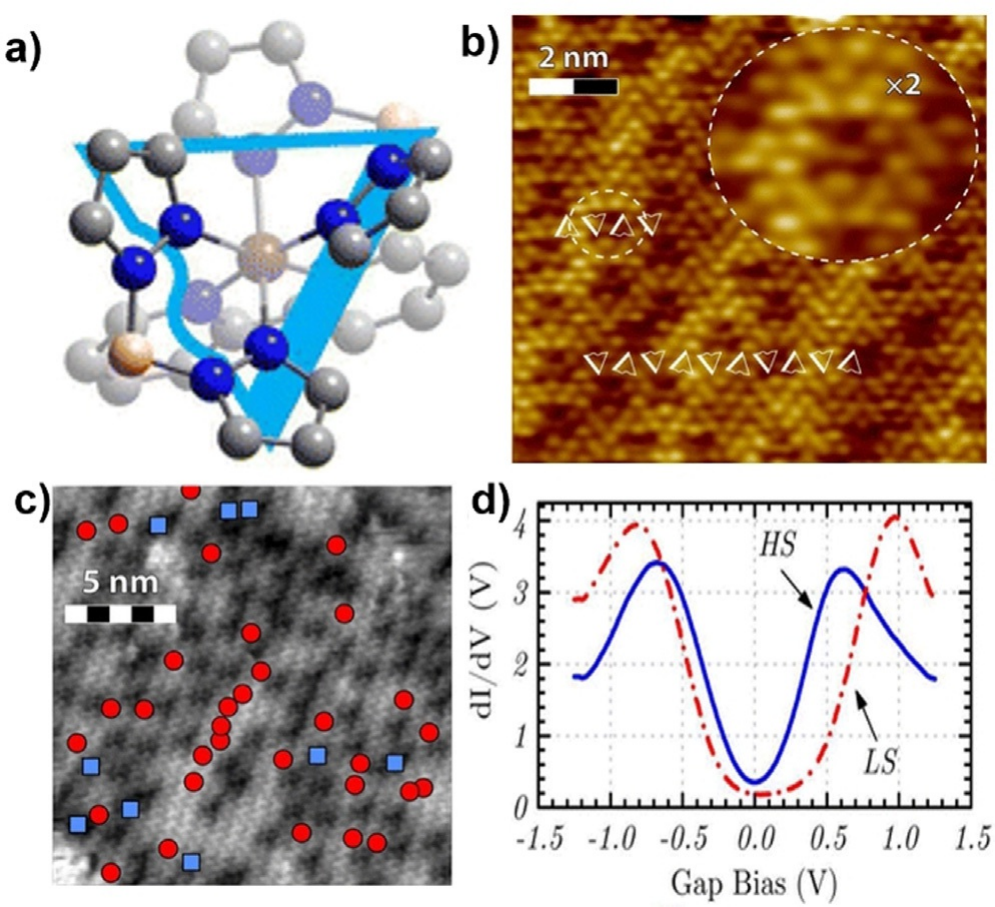
a) Top view of [Fe(H_2_B(pz)_2_)_2_(bpy)] in its bilayer orientation. The pale and bright parts of the molecule are pointing downwards and upwards, respectively; the wrinkled side of the triangle represents a fully upward‐pointing [H_2_B(pz)_2_] ligand.b) STM image (*V=*−1 V, *I*=1 nA) of the bilayer film at 131 K showing bright (LS) and depressed (HS) regions; the inset is an enlarged view of a depression. c) Conductance map (*V=*−1 V, *I*=1 nA, and *T=*131 K) of a 20×20 nm bilayer film and d) the corresponding d*I/*d*V* curves measured either above bright (LS: red circles) or depressed parts (HS: blue squares). Reproduced with permission from ref. [112]. Copyright (2013) American Chemical Society.

The spin‐state switching behavior of [Fe(H_2_B(pz)_2_)_2_(bpy)] and its functional variants has also been studied on different substrates at varying coverages. An STM study elucidated the large‐scale molecular organization (Figure 6) of [Fe(H_2_B(pz)_2_)_2_(bpy)] in bilayer films, driven by the π–π interaction between the bpy rings, on Au(111). At 131 K, a spin‐state coexistence with more dominant LS fraction (bright areas in Figure 6 b,c) was observed in the bilayer films due to surface‐mediated constraints imposed on the intermolecular interactions operating between the switching entities.^[112]^ Density functional theory (DFT) studies of the [Fe(H_2_B(pz)_2_)_2_(bpy)] bilayer on Au(111) revealed the three‐ and two‐lobed structure of the LS and HS complexes, respectively. The DFT calculations also predicted a larger HOMO–LUMO gap (HOMO=highest occupied molecular orbital and LUMO=lowest occupied molecular orbital) for the LS complex than the HS complex. Conductance (d*I/*d*V)* spectra (Figure 6 c) measured above the three‐lobed (bright) sites revealed a larger HOMO–LUMO gap than the gap observed above the two‐lobed (dark depression) sites (Figure 6 c). Based on the DFT and STS studies, the bright and dark areas in the bilayer film of [Fe(H_2_B(pz)_2_)_2_(bpy)] on Au(111) were unambiguously identified as LS and HS sites, respectively.

Contrary to the large‐scale molecular organization observed for the bilayer film, non‐uniform structural growth was observed for multilayer films thicker than 2 nm, indicating the non‐propagation of the higher‐ordered structure associated with the bilayer film. The thicker 200 nm film of the complex showed comparable spin‐state switching characteristics to the bulk phase indicating the weakening of the surface‐induced constraints at nanometer distances from the interface.^[65]^

**Figure 7:**
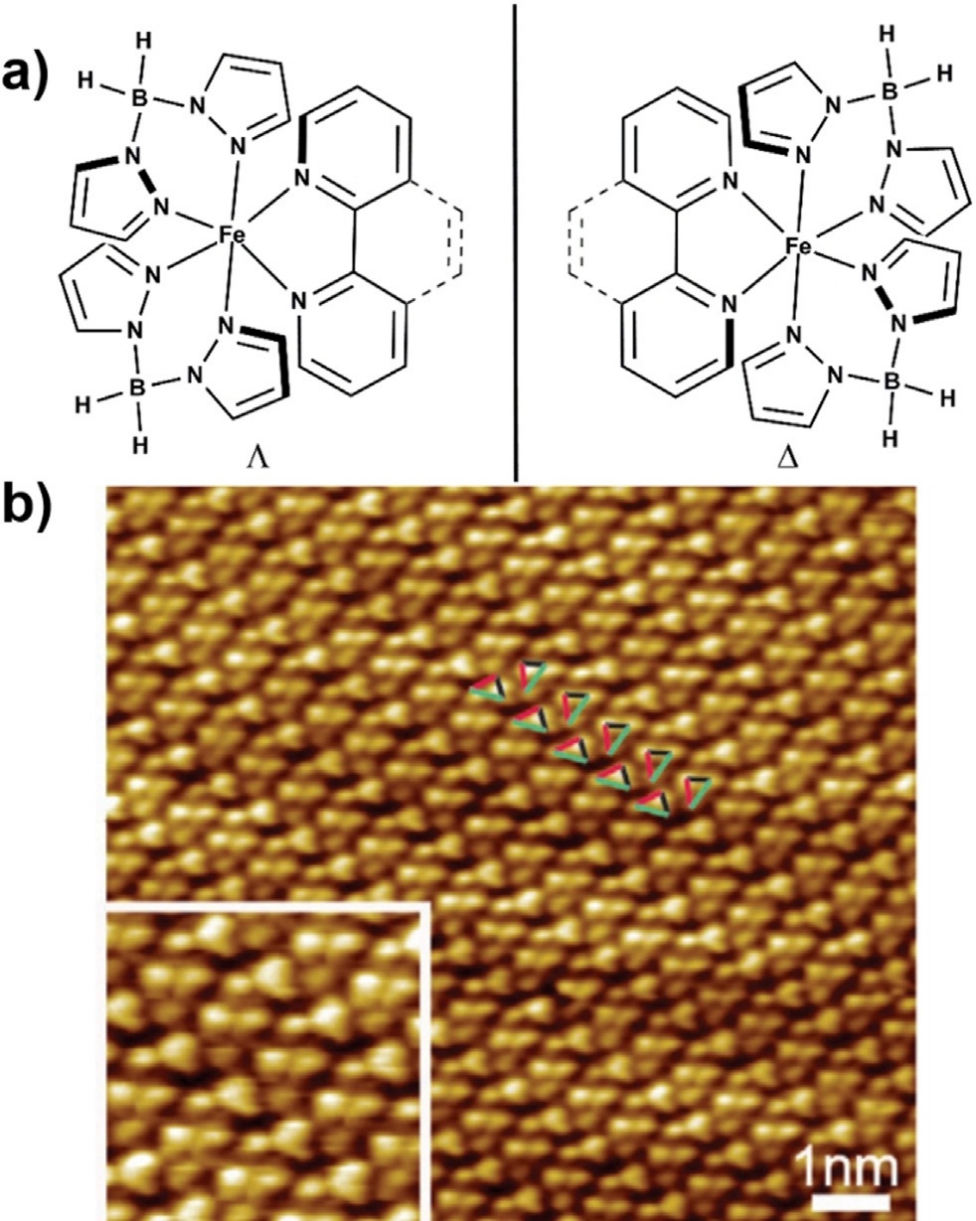
a) Λ and Δ enantiomers of the [Fe(H_2_B(pz)_2_)_2_(phen)] and [Fe(H_2_B(pz)_2_)_2_(bpy)] family of complexes and b) STM image of a bilayer domain of [Fe(H_2_B(pz)_2_)_2_(bpy)] on Au(111) at sub‐monolayer coverage showing isochiral rows with opposite handedness leading to overall racemic paving of the surface. Reproduced with permission from ref. [51]. Copyright (2016) Institute of Physics.

A facet not considered in the above studies is the enantiomeric nature (Δ or Λ) of the [Fe(H_2_B(pz)_2_)_2_(phen)] and [Fe(H_2_B(pz)_2_)_2_(bpy)] family of complexes (Figure 7). A 77 K STM study of a bilayer island of [Fe(H_2_B(pz)_2_)_2_(bpy)] on Au(111) revealed the arrangement of the complexes as isochiral rows (either Δ or Λ) in the top layer. However, the complexes in the adjacent rows are rotated 70° with respect to one another (colored triangles, Figure 7 b), which produced oppositely handed isochiral rows, thus leading to the overall racemic paving of the surface.^[51]^ The on‐surface separation of the Λ and Δ enantiomers could be achieved by subliming the complexes on intrinsically chiral substrates such as Pt(643)^[113]^ or Cu(874).^[114]^

The selective absorption of molecular systems on chiral inorganic surfaces was previously reported.^[115]^ The possible resolution of the SCO‐active racemic [Fe(H_2_B(pz)_2_)_2_(phen)] and [Fe(H_2_B(pz)_2_)_2_(bpy)] complex systems into their enantiomeric components may lead to novel chiroptical and magnetochiral spintronic components.[^116^, ^117^]

**Figure 8:**
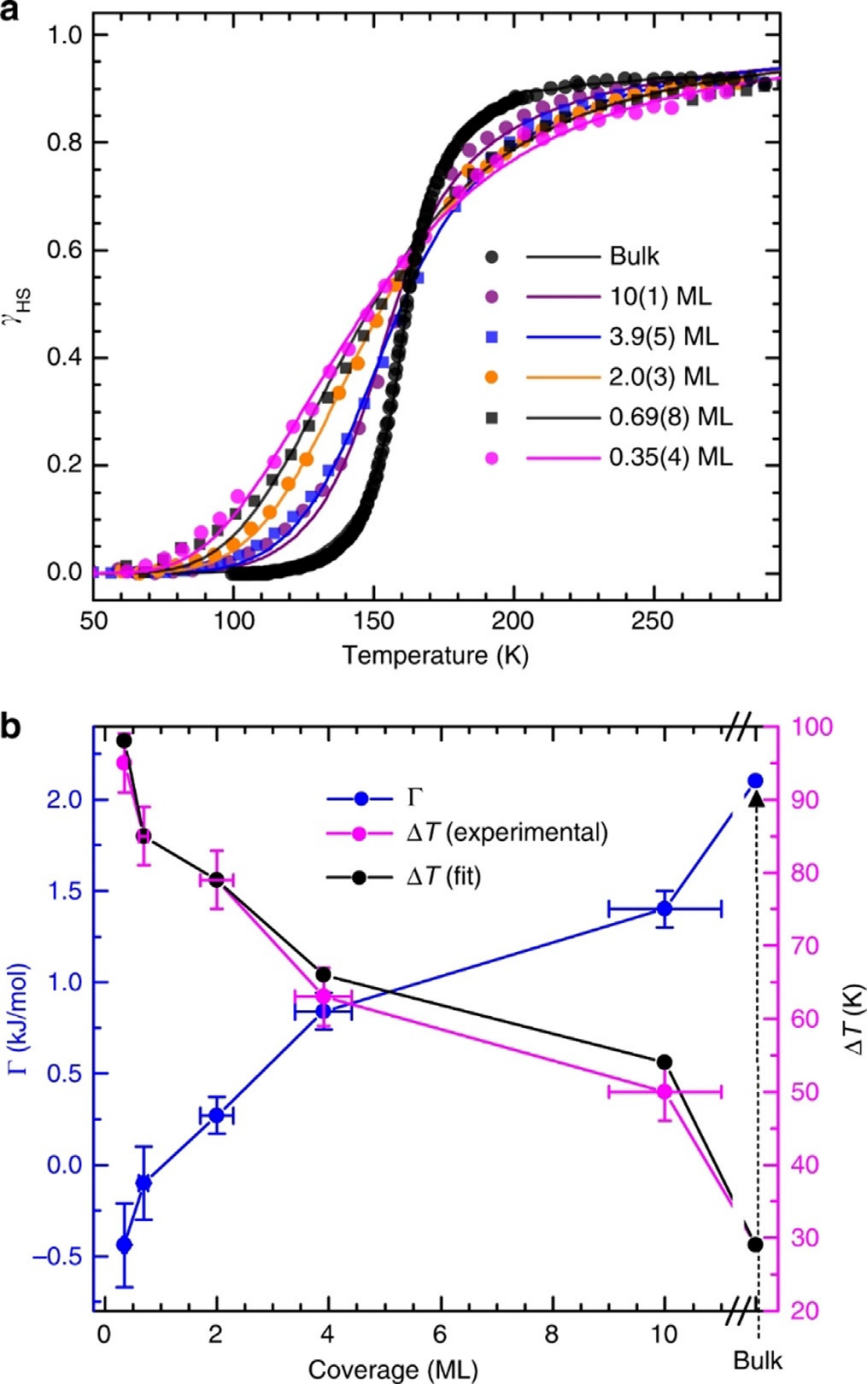
a) Coverage‐dependent SCO of vacuum‐sublimed thin films of [Fe(H_2_B(pz)_2_)_2_(bpy)] on HOPG; the HS fraction (*γ*) versus *T* curves were fitted employing the phenomenological S‐D model. b) Coverage‐dependent evolution of interaction parameter (*Γ*) and transition width; the blue and pink dots represent the interaction parameter and transition width, respectively. Note: the 10 ML thick film reached 60 % of *Γ* relative to the bulk sample. Reproduced with permission from ref. [119]. Copyright (2018) Nature Publishing Group.

Cooperative intermolecular interactions facilitating the occurrence of abrupt and first‐order SCO are a thoroughly investigated phenomena in the bulk‐state.^[118]^ In a quest to determine the size limit at which cooperativity becomes effective in thin films, thermal and light‐induced SCO of [Fe(H_2_B(pz)_2_)_2_(bpy)] on HOPG with coverages ranging from 0.35(4) to 10(1) monolayers was studied. The sub‐monolayer to multilayer thick films showed complete thermal and light‐induced SCO (Figure 8 a). The transition width of the thermal SCO curves decreased with increasing film thickness (Figure 8 a,b), evidencing increasing cooperativity with increasing film thickness. Quantitative estimation of the cooperativity was achieved by fitting the HS fraction (*γ*_HS_) versus *T* curves shown in Figure 8 a by the Slichter and Drickamer (S‐D) model [Eq. 1].

(1) $$\ln\;\left(\frac{1-\gamma_{\mathrm{HS}}}{\gamma_{\mathrm{HS}}}\right)=\frac{\Delta H\;+\;\Gamma \left(1-2\gamma_{HS}\right)}{RT}\;-\frac{\Delta S}{R}\;\;$$

Here *Γ* is the interaction parameter, Δ*H* and Δ*S* are the enthalpy and entropy changes, respectively, associated with the SCO, and *R* is the gas constant. Negative values of *Γ* obtained for 0.35 ML and 0.69 ML films indicate non‐cooperative SCO behavior, whereas the positive value of *Γ*=0.3(1) kJ mol^−1^ observed for the 2 ML film (Figure 8 b) indicates the onset of cooperative spin‐state behavior starting from the second monolayer. A subsequent increase of *Γ* with increasing film thickness indicates increased cooperativity in thicker films, with the 10 ML film showing *Γ*=1.4 kJ mol^−1^ comparable to the *Γ*=2.1 kJ mol^−1^ shown by the bulk phase.

While the thermal SCO of HOPG‐bound [Fe(H_2_B(pz)_2_)_2_(bpy)] is cooperative in nature, the LIESST‐mediated LS→HS transition of [Fe(H_2_B(pz)_2_)_2_(bpy)] on HOPG is not cooperative and molecular in nature for all the film thicknesses. Note, LIESST‐induced LS→HS switching is also molecular in the bulk phase. The stability of the light‐induced HS state of [Fe(H_2_B(pz)_2_)_2_(bpy)] at sub‐monolayer coverages on the HOPG surface was also studied in the temperature range of 8 K–40 K. Remarkably, the HS→LS relaxation behavior of the complexes in the sub‐monolayer films exhibited a stretched exponential behavior, with the relaxation rate increasing with increasing temperature. On the other hand, the HS→LS spin relaxation in the bulk sample became pronounced when *T* is close to the *T*_(LIESST)_;^[97]^ that is, in the thermally activated regime a sigmoidal relaxation behavior was observed.^[119]^ Enhanced tunneling rates and a reduced energy barrier between the metastable HS and LS states caused the increased HS→LS relaxation rate, relative to the bulk, observed for the sub‐monolayer films on HOPG.

**Figure 9:**
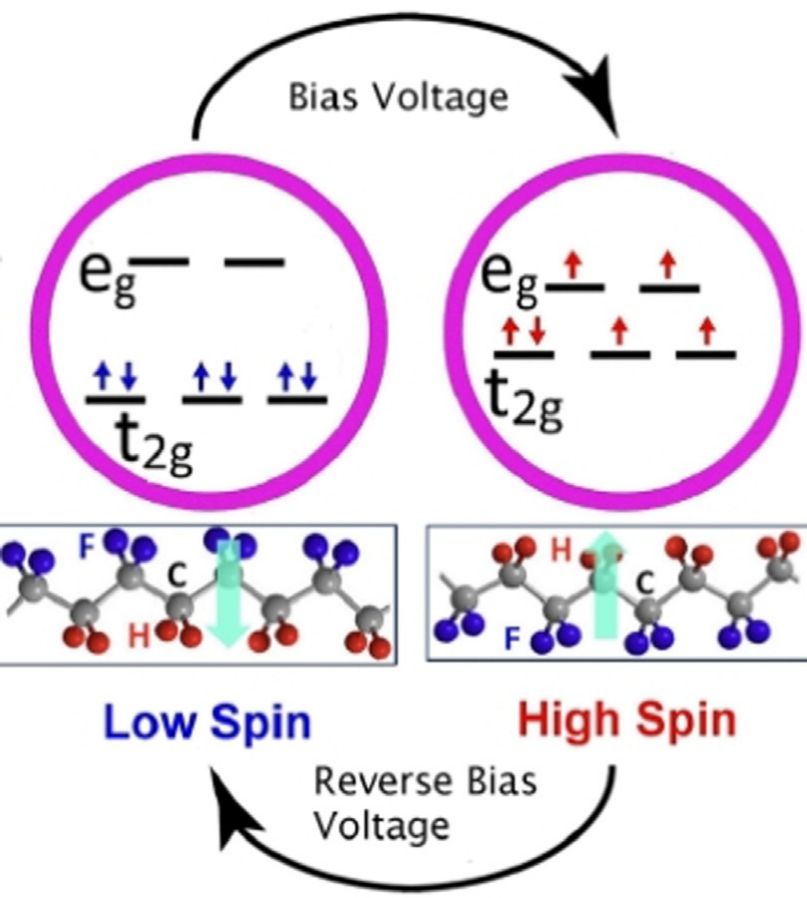
Ferroelectric polarization dependent pinning of the spin state of [Fe(H_2_B(pz)_2_)_2_(bpy)] on organic ferroelectric substrates. The complex is pinned in the LS state when the ferroelectric polarization is pointing away from the complex (left). Switching the ferroelectric polarization towards the complex pins the complex in the HS state (right). Reproduced with permission from ref. [123]. Copyright (2018) Royal Society of Chemistry.

The spin‐state switching properties of thin films of [Fe(H_2_B(pz)_2_)_2_(bpy)] were also studied on ferroelectric and magnetic oxide substrates. The SCO of the 5 nm film of the complex was also studied on dielectric oxide surfaces—Al_2_O_3_ and SiO_2_—to probe the role of electrostatic environment on determining the spin state of the complex. The 10–25 ML thick film of the complex deposited on the organic copolymer ferroelectric polyvinylide fluoride–trifluoroethylene (PVDF‐TrFE: 70‐30) showed ferroelectric polarization dependence of the spin‐state (Figure 9). When deposited on the poled “up”, that is, interfacial dipole pointing up, PVDF‐TrFE substrate, the complex remained in the HS‐state even around 100 K, which is well below the *T*_1/2_≈160 K reported for the bulk [Fe(H_2_B(pz)_2_)_2_(bpy)]. Deposition of the complex on the poled “down”, that is, interfacial dipole pointing down, ferroelectric substrate locked the complex in the LS state, well above the bulk *T*_1/2,_ around 300 K. These results evidence the ferroelectric polarization mediated suppression of thermal SCO.^[120]^ Subsequently, voltage‐controlled isothermal spin‐state switching was demonstrated for the frozen LS [Fe(H_2_B(pz)_2_)_2_(bpy)] films deposited on polyvinylide fluoride–hexafluoropropylene (PVDF‐HFP) and croconic acid ferroelectric substrates.^[121]^ On the other hand, temperature‐induced HS→LS switching of the 7 nm and 70 nm films of [Fe(H_2_B(pz)_2_)_2_(bpy)] on PMN‐PT(011) ([Pb(Mg_1/3_Nb_2/3_)O_3_]_1−*x*_[PbTiO_3_]_*x*_, *x=*0.32) is not ferroelectric polarization dependent. However, the efficiency of SOXIESST‐mediated LS→HS switching at 3 K is governed by the ferroelectric polarization of PMN‐PT(011). When the ferroelectric polarization of the PMN‐PT(011) points toward the surface, the SOXIESST mediated LS→HS switching proceeded with a larger magnitude than that of the opposite polarization.^[122]^

Magnetic‐field‐mediated spin reversal in a 10 nm thick film of [Fe(H_2_B(pz)_2_)_2_(bpy)] on NiCo_2_O_4_ and La_0.67_Sr_0.33_MnO_3_ (LSMO) magnetic oxide substrates was also demonstrated at RT. Soft X‐ray irradiation of the largely LS‐[Fe(H_2_B(pz)_2_)_2_(bpy)] on NiCo_2_O_4_ and LSMO substrates induced LS→HS switching; application of an oscillating external magnetic field facilitated the relaxation of the exited HS‐state to the LS‐state. The magnetic‐field‐mediated HS→LS switching was observed for [Fe(H_2_B(pz)_2_)_2_(bpy)] even under X‐ray irradiation on NiCo_2_O_4_, whereas X‐ray fluence needs to be switched off to facilitate HS→LS switching of [Fe(H_2_B(pz)_2_)_2_(bpy)] on LSMO. This surface dependence of the magnetic‐field‐induced HS→LS relaxation evidences the magnetic coupling between [Fe(H_2_B(pz)_2_)_2_(bpy)] and NiCo_2_O_4_/LSMO substrates.^[123]^

The spin‐state of a several nm thick [Fe(H_2_B(pz)_2_)_2_(bpy)] film is locked in the LS‐state when it is deposited on SiO_2_/Al_2_O_3_ dielectric substrate; soft X‐ray irradiation of the films induced LS→HS switching at RT.^[124]^ The 900 nm (Δ*T*=15 K) and 300 nm (Δ*T*=5 K) films of [Fe(H_2_B(pz)_2_)_2_(bpy)] on Al_2_O_3_, in contrast to the bulk, showed hysteretic SCO mediated by [Fe(H_2_B(pz)_2_)_2_(bpy)]/Al_2_O_3_ interfacial interactions.^[125]^

**Figure 10:**
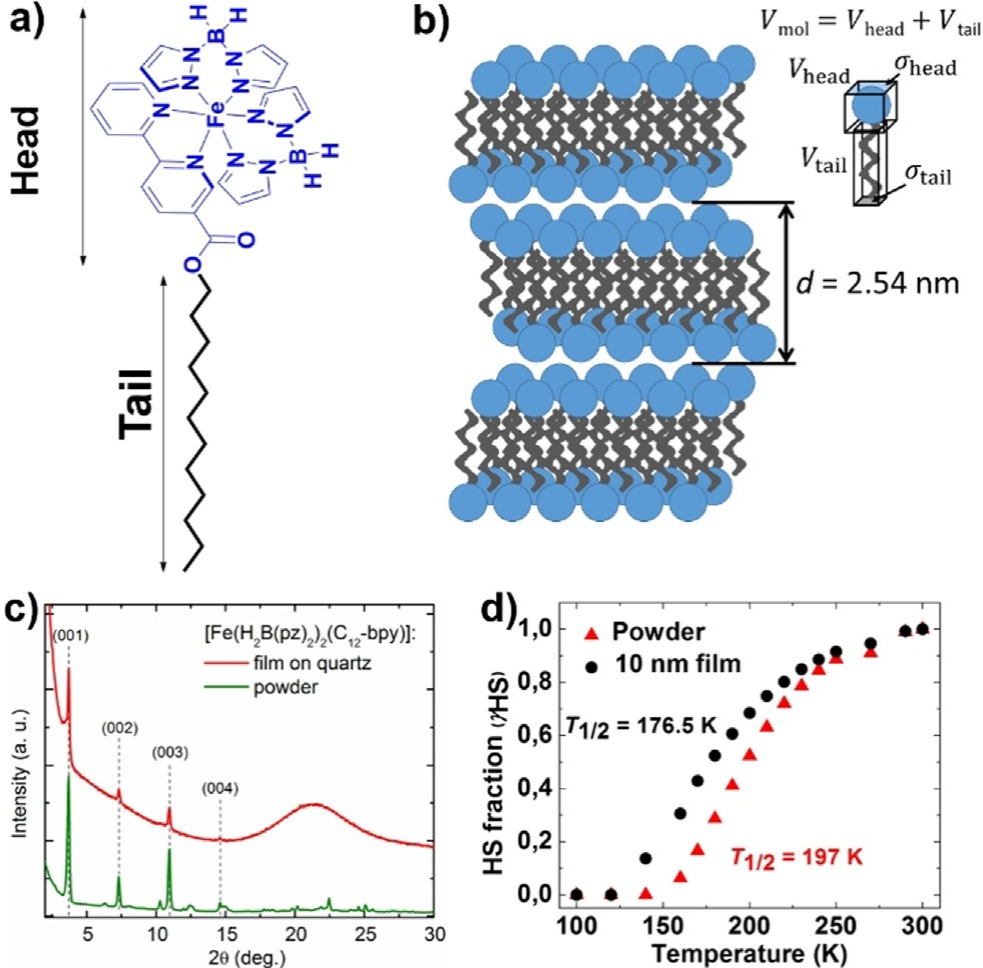
a) Molecular structure of the pseudo‐amphiphile‐like SCO complex [Fe(H_2_B(pz)_2_)_2_(L^7^)]. b) Schematic representation of lamellar self‐organization of the complex. c) X‐ray diffraction pattern of a 10 nm [Fe(H_2_B(pz)_2_)_2_(L^7^)] film on quartz substrate compared to the powder reference. d) Temperature dependence of HS fraction of the powder and thin‐film forms of [Fe(H_2_B(pz)_2_)_2_(L^7^)] evaluated by XAS.

The above studies establish the importance of factors such as the nature of the surface, interfacial electronic structure, and intermolecular interactions in affecting the SCO characteristics of the sublimed thin‐film architectures. However, direct control of the intermolecular interactions, a governing factor in dictating SCO in the bulk phase, is a rather difficult problem to tackle at the nanoscale, especially on surface‐bound thin films. A molecular self‐assembly strategy was invoked to copy the self‐organization of the bulk phase in the thin‐film state. Functionalization of the parent complex system [Fe(H_2_B(pz)_2_)_2_(bpy)] with a dodecyl (C_12_) alkyl chain yielded a classical amphiphile‐like functional and vacuum‐sublimable charge‐neutral Fe^II^ complex, [Fe(H_2_B(pz)_2_)_2_(L^7^)] (Figure 10 a).

Remarkably, the complex self‐organized as lamellar bilayer structures both in the bulk and sublimed films (Figure 10 b), as evidenced by the X‐ray studies shown in Figure 10 c. The bulk powder and 10 nm thin‐film forms of the complex showed comparable SCO behavior (Figure 10 d) mediated by similar lamellar‐bilayers‐like self‐assembly in both bulk and thin‐film states. Importantly, the sublimation propensity of [Fe(H_2_B(pz)_2_)_2_(L^7^)] demonstrates the possibility of subliming functional‐group‐appended SCO complexes, encouraging the study of functional SCO complexes on the surface for applications.^[105]^

### 3.3 Fe[HB(3,5‐(Me)_2_pz)_3_]_2_

In the bulk‐state, Fe[HB(3,5‐(Me)_2_pz)_3_]_2_ undergoes bistable SCO with *T*_1/2_≈190 K and Δ*T*=31 K. Unlike the previously discussed systems forming good‐quality thin films, grainy films were obtained upon sublimation of Fe[HB(3,5‐(Me)_2_pz)_3_]_2_ onto a surface, with roughness and crystallite size increasing with increasing film thickness. Incomplete SCO with the remnant and SCO‐inactive metastable HS portion was observed for 8.02 μm and 130 nm films on quartz. Annealing of the films at 400 K resulted in near‐complete spin‐state switching with *T*_1/2_=152 K and Δ*T*=17 K.^[94]^ X‐ray diffraction studies of the as‐sublimed 8.02 μm film revealed peaks at 2*θ*=9.98° and 10.1° corresponding to the tetragonal and triclinic polymorphs, respectively. This indicates the coexistence of tetragonal and triclinic polymorphs in the as‐sublimed films, in contrast to the bulk sample that crystallizes in the triclinic space group. Thermal annealing of the films resulted in the disappearance of the peak (2*θ*=9.98°) corresponding to the tetragonal phase and retention of the peak (2*θ*=10.1°) associated with the triclinic phase. Thus, the observed similarities in the SCO of thermally annealed films and bulk samples are due to the triclinic space group associated with the samples; the coexistence of tetragonal and trigonal phases rendered SCO of the as‐prepared films incomplete. A 570 nm thick film of Fe[HB(3,5‐(Me)_2_pz)_3_]_2_ on Si showed SCO with *T*_1/2_ and Δ*T* in the range observed for the 130 nm post‐annealed film on quartz.^[66]^

**Figure 11:**
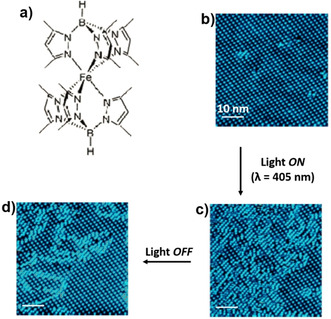
a) Molecular structure of Fe[HB(3,5‐(Me)_2_pz)_3_]_2_. b–d) STM images (*T*=4.6 K, *V=*0.3 V, and *I*=20 pA) of the b) as‐prepared S_1/3_ superstructure of Fe[HB(3,5‐(Me)_2_Pz)_3_]_2_ at sub‐monolayer coverage on Au(111), c) *S*_1/2_ superstructure obtained after irradiating the S_1/3_ superstructure with blue light (*λ*=405 nm) for 9.45 h, and d) relaxed state obtained 9.45 h after the light irradiation had been stopped. Reproduced with permission from ref. [126]. Copyright (2016) Nature Publishing Group.

In contrast to the near‐complete SCO of the annealed films, a sub‐monolayer film of Fe[HB(3,5‐(Me)_2_pz)_3_]_2_ in direct contact with Au(111) surface showed an incomplete SCO with *T*_1/2_≈154 K and Δ*T*=7 K. A spin‐state coexistence, with one‐third of the molecules in HS state, was observed below 100 K.^[82]^ STM analyses of sub‐monolayer films of Fe[HB(3,5‐(Me)_2_pz)_3_]_2_ at 4.6 K revealed stable long‐range‐ordered self‐organization of the complex molecules up to a domain size of 200×200 nm^2^. The mixed‐spin‐state domains are constituted of an *S*_1/3_ superstructure (unit cell) made up of one HS molecule and two LS molecules. Blue‐light (*λ*=405 nm) irradiation, at 4.4 K, of the *S*_1/3_ superstructure, induced the formation of another *S*_1/2_ superstructure, 10×10 nm^2^, with a time constant of 114±8 min, indicating the LIESST‐active nature of the complex in direct contact with Au(111). The *S*_1/2_ superstructure is made of equal proportions of HS and LS molecules, indicating a partial LIESST‐mediated LS‐HS switching. When the blue‐light illumination was stopped, the *S*_*1/2*_ superstructure relaxed back to the *S*_1/3_ superstructure with a relaxation time of 131±5 min (Figure 11).^[126]^ The bright HS molecules of [Fe[HB(3,5‐(Me)_2_pz)_3_]_2_ showed no Kondo effect at 4.6 K, indicating the weak coupling between the HS complex and the Au(111) substrate. An unambiguous spin‐state identification was achieved by performing inelastic tunneling spectroscopy on the individual complex: LS and HS molecules appeared as dark and bright spherical structures, respectively.

### 3.4 [Fe(HB(trz)_3_)_2_]

Complex [Fe(HB(trz)_3_)_2_] was studied as 20–200 nm thick films on fused silica, crystalline silicon (100), and polycrystalline Au substrates. The as‐prepared amorphous films crystallized upon water vapor annealing (relative humidity=75–80 %) at RT. The crystalline films showed thickness‐independent first‐order SCO (*T*_1/2_=338 K) as the bulk sample.^[74]^ The as‐prepared 100–200 nm thick films of the complex on fused silica substrate were solvent annealed to establish the nature of solvent in affecting the crystallinity, morphology, and SCO. Water, diethyl ether, acetone, and ethanol solvents capable of accepting hydrogen bonds yielded highly oriented crystalline films, which showed an abrupt and complete SCO with *T*_1/2_=336 K. Films annealed with dichloromethane (not a hydrogen‐bond acceptor) showed incomplete SCO due to the poor crystallinity of the film. Among the solvents studied, water yielded high‐quality and continuous films. Water vapor annealing of the films above and below 72 % relative humidity yielded crystalline and semicrystalline films, respectively. Unlike the crystalline films, the semicrystalline films exhibited incomplete SCO. The effect of thickness reduction on the SCO of microcrystalline thin films of [Fe(HB(trz)_3_)_2_], deposited on fused silica substrate, was probed in the 200–45 nm range. A ≈3 K elevation of the *T*_1/2_ value, that is, stabilization of the LS phase, is observed upon thickness reduction. The increase in the surface energy of the HS‐state relative to the LS state by 5 mJ m^−2^ caused the shift in the transition temperature in the 45 nm film.^[73]^

Light irradiation of the SCO solids with femtosecond laser pulses leads to an out‐of‐equilibrium dynamical spin‐state switching process, which involves a sequence of three consecutive steps, namely, photo‐, elastic, and thermal switching at distinct ps, ns, and μs timescales, respectively.[^88^, ^127^, ^128^] The photo‐induced step involves ultrafast electronic and structural reorganization, that is, LS→HS switching and the associated volume change, at the molecular scale. The consequent noninstantaneous elastic step causes a large‐scale volume change of the lattice due to strain propagation throughout the lattice. The thermal switching process is triggered by heat diffusion, which increases the average temperature of the lattice. Since both the strain wave propagation and heat diffusion take place at shorter timescales in nanoscale systems, size reduction is a promising route to achieve ultrafast switching dynamics.^[129]^ Further, on the bulk scale, the finite laser penetration depth (*δ*=50 μm at 570 nm) produces an inhomogeneous distribution of photoswitched molecules, which induce delayed dynamic responses. The bulk inhomogeneous effects can be suppressed in nanoscale objects and thin films with thicknesses less than the laser penetration depth. Moreover, the nano‐ and microsecond dynamical responses associated with strain propagation and heat diffusion, respectively, can be fine‐tuned by varying the film thickness.^[130]^

**Figure 12:**
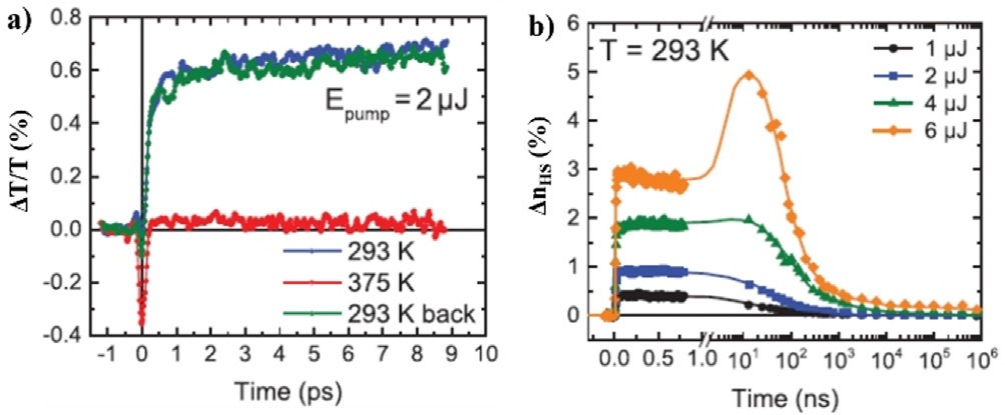
Photo‐response of the 100 nm thin film of [Fe(HB(trz)_3_)_2_] on the ps–ns time range after a femtosecond laser pulse irradiation at *λ*=570 nm. a) Time evolution of the relative change of the optical transmission (Δ*T/T*) at *λ*=320 nm at 293 K and 375 K after laser pulse irradiation and b) time evolution of the HS fraction (Δ*n*_HS_) after laser pulse irradiation with excitation energies in the range of 1 μJ–6 μJ.

In this context, to understand the evolution of the dynamical properties of the light‐induced spin‐state switching in the nanometer regime, RT spin‐state switching dynamics in water solvent annealed thin films of [Fe(HB(trz)_3_)_2_] (50 nm, 100 nm, and 150 nm) deposited on fused silica substrates were studied employing femtosecond pump–probe optical absorption spectroscopy. The thin films were irradiated at *λ*=570 nm corresponding to the d–d transition band of the complex in the LS state. After irradiation (*λ*=570 nm) of the 100 nm thick film with a femtosecond laser pulse, the time evolution of the relative change of the optical transmission (Δ*T/T*) in the charge‐transfer region of the LS‐state (*λ*=320 nm) on the ps timescale was probed at 293 K (LS) and 375 K (HS) (Figure 12 a). At 293 K, a photo‐induced signal due to the light‐induced LS→HS switching of a small number of molecules was observed (the blue and green curves in Figure 12 a), whereas photo‐irradiation of the same area of the film above the SCO transition temperature, 375 K, produced no detectable photo‐induced signal (the red curve in Figure 12 a). The photo‐induced transition shown in Figure 12 a involves two different processes occurring at *τ* ^1^=171±8 fs and *τ* ^2^=2.4±0.4 ps timescales. The shorter and longer time constants are associated with the intersystem‐crossing‐mediated population of the HS‐state and the vibrational relaxation of the HS complexes in the higher vibrational levels of the HS potential well, respectively.

Probing of the photo‐induced dynamics at RT in the 100 nm thick film on a longer 100 ps to ms temporal range evidenced different photoresponses (Figure 12 b). For laser pulse energies below 4 μJ, the HS complexes return to the LS‐state through a single‐step relaxation process within 100 ns. A pronounced increase of the fraction of HS (Δ*n*_HS_) complexes in the 20–40 ns time window was observed above a threshold excitation power of 6 μJ (Figure 12 b). The two‐step response was also observed in the 150 nm thick film, whereas such a response was absent in the 50 nm thick film. The photo‐response occurring in the ns regime is attributed to a thermally activated LS→HS switching process, which delays the decay of the photo‐induced HS species. Overall, a sub‐picosecond intramolecular LS→HS photoswitching and a thermally activated LS→HS switching in the ns regime is observed in the 150 nm and 100 nm films. Efficient heat transfer to the substrate in the 50 nm film prevented the occurrence of the second switching step and accelerated the recovery of the spin‐state equilibrium, giving evidence of the size‐reduction effects on the photo‐induced dynamics of spin‐state switching at the nanoscale, especially in thin‐film architectures.^[130]^

### 3.5 Other Systems

The complex [Fe(dpepd)(NCS)_2_], deposited as a sub‐monolayer (0.8 ML) film on HOPG, underwent gradual and fully reversible thermal SCO with *T*_1/2_=235(6) K. On a comparative scale, the sub‐monolayer film of the complex showed more gradual SCO with a lower transition temperature than the bulk phase, which exhibited SCO with *T*_1/2_=251(3) K. The study was the first example showing the utility of a carbon‐based substrate in preserving the SCO behavior of molecules in direct contact with a substrate.^[90]^ A 50 nm film of [Fe(qnal)_2_] on gold showed temperature‐dependent (*T*_1/2_=210 K) and LIESST‐ and SOXIEEST‐mediated SCO. The SCO of [Fe(qnal)_2_] in thin‐film proceeded at a lower temperature relative to the bulk.^[72]^

A 40 monolayer thick film of [Fe(pypyr(CF_3_)_2_)_2_(phen)] on layered metallic 1T‐TiTe_2_ showed *T*_1/2_=330 K, which is significantly lower than the transition temperature (*T*_1/2_=390 K) observed for the bulk complex. In contrast to the bulk complex (*T*_LIESST_≈2 K), the 40 ML film showed light‐induced LS→HS switching below 100 K: the HS‐state is more stable in the thin‐film‐state than in the bulk‐state.^[95]^ At sub‐monolayer coverage, fragmentation of [Fe(pypyr(CF_3_)_2_)_2_(phen)] occurred on metallic substrates (Co/Cu(100), Au(111), and graphene/Ni(111)). A significantly reduced fragmentation of the complex occurred on semiconducting (WSe_2_ and HfS_2_: 5 %) and semimetal (HOPG: 2 %) substrates. Insertion of graphene as a decoupling layer between Ni(111) and [Fe(pypyr(CF_3_)_2_)_2_(phen)] reduced fragmentation: about 70 % of the complexes remained intact. The sub‐monolayer films deposited on semiconducting and semimetal substrates showed light‐induced LS→HS switching at temperatures below 100 K. The above results demonstrate the role of the density of states near the Fermi level in determining the stability of [Fe(pypyr(CF_3_)_2_)_2_(phen)] in direct contact with a substrate.^[131]^

STM analysis of a sub‐monolayer film of [Fe(pap)_2_]^+^ on Au(111) showed the presence of an intact complex cation, ClO_4_^−^ anions, and fragments of [Fe(pap)_2_]^+^; attempted STM‐tip‐induced switching of intact [Fe(pap)_2_]^+^ was not successful.^[83]^

In short, at sub‐monolayer coverage, the coexistence of spin‐states and the freezing of SCO were reported for complexes studied on metallic surfaces, especially on Au(111). More specifically, the molecular structure of [Fe(phen)_2_(NCS)_2_] remained intact on Au(111) due to the chemisorbing NCS ligand. However, chemisorption blocked the SCO of [Fe(phen)_2_(NCS)_2_] at sub‐monolayer coverage. On the other hand, the SCO of the [Fe(H_2_B(pz)_2_)_2_(L)] (L=phen or bpy) family of complexes is also frozen on metallic substrates even though the adsorption of the complexes on metallic substrates was better described as physisorption, resulting in weak electronic coupling between the complex and substrate. Thus, the reported partial disintegration of the [Fe(H_2_B(pz)_2_)_2_(L)] complexes upon deposition on Au(111) and surface‐induced constraints caused the spin‐state freezing of the complexes. The intact nature of the homoleptic complex [Fe[HB(3,5‐(Me)_2_pz)_3_]_2_] on Au(111) and the absence of the expected Kondo resonance demonstrates the weak electronic coupling between [Fe[HB(3,5‐(Me)_2_pz)_3_]_2_] and Au(111). Thus, the observed spin‐state coexistence of [Fe[HB(3,5‐(Me)_2_pz)_3_]_2_] at sub‐monolayer coverage on Au(111) is due to the epitaxial constraints between [Fe[HB(3,5‐(Me)_2_pz)_3_]_2_] and the surface.

The role of the continuum of metallic surface states in blocking SCO of an absorbed complex was further demonstrated by studying the SCO of complexes on electronically passive carbonaceous HOPG and semimetallic substrates. The occurrence of complete thermal and light‐induced SCO of [Fe(phen)_2_(NCS)_2_] and [Fe(L^5^)_2_(NCS)_2_] on HOPG at sub‐monolayer coverage testifies to the active role of the metallic surface in blocking SCO. In a closer scenario, the spin‐state switching was deliberately restored by decoupling the underlying metallic substrate (Cu(100)) and [Fe(phen)_2_(NCS)_2_] by sandwiching an insulating layer (CuN) between them.

The close resemblance between the SCO of the bulk and multilayer films of most of the sublimable SCO complexes further demonstrates the role of the interfacial electronic structure in blocking SCO of the molecules in direct contact with the metallic substrates. Thus, it is conclusive that the spin‐state coexistence of the SCO complexes at sub‐monolayer coverage on metallic surfaces primarily depends on the magnitude of the electronic coupling between the SCO molecule and the substrate and the stability of the complex under study.

Another important aspect is the lack of control associated with the thin‐film growth leading to the difference in the intermolecular interactions in the thin‐film state relative to the bulk state. Since intermolecular interactions are a dominant factor governing the nature of SCO, achieving the same molecular organization both in bulk and thin‐film forms may manifest as a comparable SCO behavior. We have elucidated the utility of this concept by tethering the parent [Fe(H_2_B(pz)_2_)_2_(bpy)] with a self‐assembly‐directing alkyl chain substituent. Although the results are encouraging, the presence of the insulating alkyl chain is a potential hindrance to realizing device architecture. However, this proof of concept could be extrapolated towards the design of device‐suitable molecules such as hybrid sublimable SCO complexes tethered with organic semiconductors and self‐assembly‐directing groups.

From the molecular structure point of view, designing homoleptic complexes featuring bulky ligands with donor atoms less likely to interact with the surface may be a strategy to promote the spin‐state switching of SCO complexes in direct contact with the surface. Note the heteroleptic nature of the [Fe(H_2_B(pz)_2_)_2_(bpy)] complex and the affinity of the bpy/phen ligand to Au(111), which facilitates fragmentation of the complex upon direct interaction with the surface, and the intact nature of the homoleptic [Fe[HB(3,5‐(Me)_2_pz)_3_]_2_] complex on the Au(111) surface. Careful molecular design and the right choice of the substrate is the need of the hour to realize switchable devices based on SCO complexes.

## 4 Device Architectures

Ever since the first proposal of a molecular rectifier by Aviram and Ratner in 1974,^[132]^ the charge transport characteristics of molecular materials have been studied, aiming the bottom‐up fabrication of molecular electronic devices.[^13^, ^133^, ^134^, ^135^, ^136^] In a later development, the report of giant magnetoresistance (GMR)[^137^, ^138^] fueled the growth of molecular spintronics, which uses both charge and spin degrees of freedom to produce power‐efficient devices. In molecular electronics and spintronics, the charge and spin transport, respectively, characteristics of single or ensembles of molecules are studied by depositing them on suitable substrates. Such molecular deposition on metallic electrodes leads to the formation of a hybrid spinterface. While the spin‐transport characteristics upon electron injection from the bottom electrode are governed by the spinterface in spintronic junctions,[^46^, ^47^, ^139^, ^140^, ^141^] the transport in molecular electronic junctions is determined by the orientation of frontier molecular orbitals with respect to the work function of the metallic electrode.[^135^, ^142^] In spintronics junctions composed of magnetic molecules, the molecules are sandwiched between electrodes, and the spin‐transport characteristics are largely determined by the magnetic state of the molecule.[^143^, ^144^, ^145^, ^146^, ^147^] Organic radicals, single‐molecule magnets (SMMs), and SCO complexes are prime examples of magnetic molecules studied in spintronic junctions.[^148^, ^149^, ^150^] The bistable SCO complexes are more advantageous for the fabrication of molecular electronic/spintronics devices due to their switchable magnetic characteristics effected by device‐suitable electric field or light stimulus.[^151^, ^152^, ^153^, ^154^, ^155^, ^156^, ^157^] At the molecular scale, a spin‐state change leads to a change in the electronic band gap, and the band gap is smaller in the HS‐SCO complexes than in their LS counterparts. Moreover, the relative orientation of the frontier molecular orbitals (FMOs) with respect to the work function of the electrode is also modified upon SCO, which in turn controls the charge‐transport characteristics of the molecular and thin‐film SCO junctions. These electronic effects occurring upon SCO manifest as conductance switching, enabling the construction of SCO‐based switching and memory elements. Another interesting aspect of the SCO‐based metal/SCO molecule/metal junction is the ability of the HS‐state to mediate spin‐polarized transport upon charge injection from the nonmagnetic metallic electrode. Thus, the HS molecule could act as a spin valve/filter—a fundamental spintronics element.[^158^, ^159^, ^160^] Apart from the electronics and spintronics applications, the elastic nature of the SCO complexes is utilized to build nanoscale actuators and resonators.^[11]^ A concise outlook of device architectures based on sublimable SCO complexes is presented in the following sections.

### 4.1 Single‐Molecule Devices

**Figure 13:**
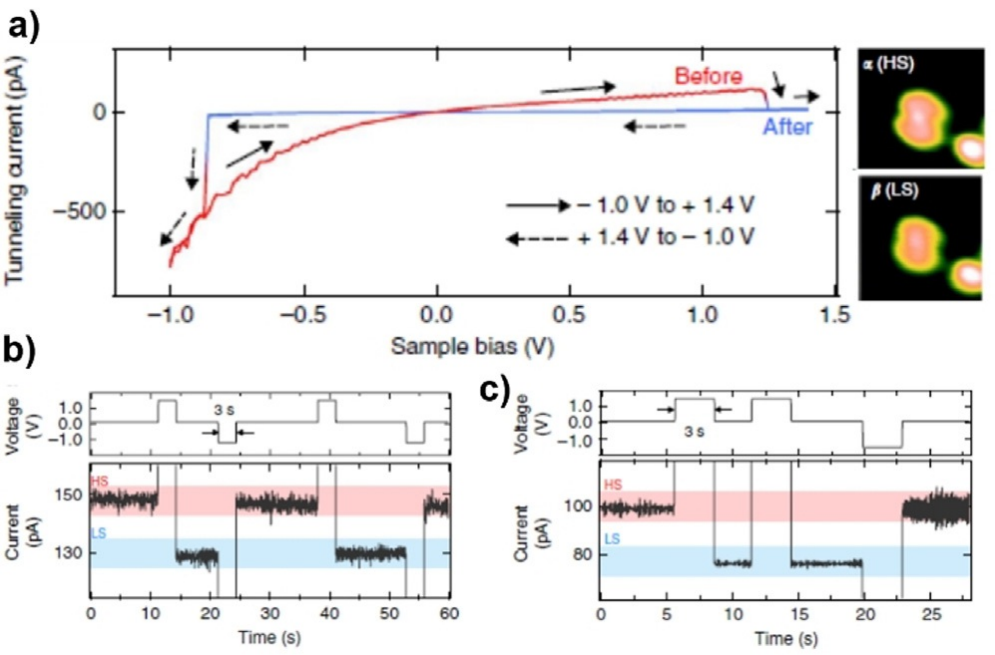
a) *I–V* characteristics of single [Fe(phen)_2_(NCS)_2_] molecules on the CuN surface. At an applied voltage of +1.2 V, the spin state of the molecule is switched from HS to LS, leading to an abrupt decrease in current; at around −0.8 V, the LS state is converted back to the HS state. The hysteretic behavior at the intermediate voltages confer memory effect to the system. b) Demonstration of the stable and reversible switching nature of the junction; application of +1.5 and −1.2 V pulses resulted in the population of LS and HS states of the complex, respectively, with accompanied current (*I*) variation. c) Application of the first positive voltage pulse switched the HS state to the LS state, whereas the application of a second positive voltage pulse effected no spin state, indicating the deterministic nature of the switching. Reproduced with permission from ref. [77]. Copyright (2012) Nature Publishing Group.

A single‐molecule STM and STS study elucidated combined spin and conduction switching of [Fe(phen)_2_(NCS)_2_] on CuN. The SCO of [Fe(phen)_2_(NCS)_2_] in direct contact with the Cu(100) surface is quenched. Decoupling [Fe(phen)_2_(NCS)_2_] and Cu(100) by adding a CuN intermediate layer facilitated external control over the spin‐state of [Fe(phen)_2_(NCS)_2_]. The voltage‐induced switching of [Fe(phen)_2_(NCS)_2_] on the CuN surface is performed by placing the STM tip at the center of the two‐lobed HS molecule, where the Fe atom is situated. Cyclic *I*(*V*) measurements in the −0.8 V to +1.4 V range revealed abrupt changes in the *I*(*V*) curve as depicted in Figure 13 a.

Remarkably, the cyclic *I*(*V*) profiling resulted in hysteretic behavior at intermediate voltages. The co‐existence of spin‐state‐dependent conductance (resistance) variation along with hysteresis (memory) renders the system a memory resistor or memristor. The observation of a stable and reproducible *I–V* response over several cycles indicates the deterministic nature of the memristor. The system is addressed, that is, the reading of the information stored in the memristor, by applying positive or negative bias pulses.^[77]^ Another interesting study demonstrated memristive behavior of [Fe(pap)_2_]^+^ based on its robust, selective, and high‐yield spin‐state switching on a Cu_2_N/Cu(100) surface.^[161]^

### 4.2 Thin‐Film Devices

Probing the spin‐state‐dependent conductance of SCO‐active thin films is necessary to realize large‐area electronics/spintronics elements. The first‐ever study in this direction reported electrical conductance characteristics of a Au/[Fe(phen)_2_(NCS)_2_] (240 nm)/Au junction. At RT, the current–voltage (*I–V*) characteristics of the junction, composed of HS [Fe(phen)_2_(NCS)_2_] molecules, is Ohmic at low bias voltages. Above 1.4 V, the conductance belongs to the space‐charge‐limited current (SCLC) regime with a mobility (*μ*)=6.53×10^−6^ cm^2^ Vs^−1^, which is comparable to the mobility of small organic molecules. The RT transport is mediated by the HS‐state due to the close proximity of the HOMO level of HS [Fe(phen)_2_(NCS)_2_] to the work function of Au (5.1 eV); the temperature dependence of the electrical conductance of the film was not studied.^[69]^

The optoelectronic transport characteristics of 10 nm, 30 nm, and 100 nm films of [Fe(H_2_B(pz)_2_)_2_(phen)] embedded between indium tin oxide (ITO) and aluminum (Al) electrodes were probed. Variable‐temperature *I–V* measurements of the ITO/[Fe(H_2_B(pz)_2_)_2_(phen)]/Al junctions, in the absence of light irradiation, showed activationless tunneling conductivity for the 10 nm thick SCO film. On the other hand, the thicker 30 nm and 100 nm junctions exhibited diode‐like rectifying characteristics and bulk‐limited thermally activated currents. Remarkably, the conductance of the 30 nm film at a constant bias of 5 V increased with increasing temperature in the 100 K to 293 K range, which is ascribed to the occurrence of gradual thermal SCO in the junction. Visible‐light irradiation of the 10 nm junction at 5 K resulted in a 7 % reduction of current intensity mediated by the LIESST‐induced LS→HS switching of the film. Successive OFF–ON–OFF irradiation cycles at 5 K led to no change in the current intensity due to the persistence of the metastable HS state at 5 K. Heating the device to 100 K restored the high‐conductance‐state due to the relaxation of the metastable HS‐state to the LS‐state. *I–V* measurements performed by irradiating the junctions at 100 K, far above the *T*_(LIESST)_=46 K of [Fe(H_2_B(pz)_2_)_2_(phen)], resulted in a negligible change of the conductance: LIESST‐induced conductance modulation at 5 K is elucidated. Note, at 5 K the thin films of [Fe(H_2_B(pz)_2_)_2_(phen)] in the LS‐state are more conductive than in the HS‐state. Similar light‐induced conductance variations were observed for the thicker 30 nm and 100 nm films at 5 K.^[162]^

The spin‐state dependence of the junction resistance in the thicker ITO/[Fe(H_2_B(pz)_2_)_2_(phen)]/Al optoelectronic junctions at 5 K was governed by the carrier hopping rates. The hopping rate increased with increasing phonon frequencies. On the other hand, the LS→HS switching results in the decrease of vibrational frequencies due to the elongation of metal–ligand bonds. The reduced frequencies associated with the HS [Fe(H_2_B(pz)_2_)_2_(phen)] films manifest as reduced charge carrier hopping rates; thereby decreased conductivity was observed upon LIESST‐mediated LS→HS switching at low temperatures. A point noteworthy here is that the reduction in the HOMO–LUMO gap and the energetic proximity of the FMOs to the Fermi level of the electrodes seem to play no role in governing the transport properties of the ITO/[Fe(H_2_B(pz)_2_)_2_(phen)]/Al optoelectronic junctions. In a nutshell, the current switching in the thicker ITO/[Fe(H_2_B(pz)_2_)_2_(phen)]/Al optoelectronic junctions is not directly related to the change in electronic spin states, rather the conductivity change is due to the coupling of electronic states with the phonon density of states, which affect the carrier hopping rates.^[162]^

The spin‐state switching mediated switching of the conductance mechanism, that is, from tunneling to hopping, and the more conductive nature of the HS [Fe(H_2_B(pz)_2_)_2_(phen)] films, relative to the LS complex, was established for the ^TS^Au/[Fe(H_2_B(pz)_2_)_2_(phen)]/EGaIn (^TS^Au=template‐stripped gold substrate, EGaIn=eutectic gallium–indium) junctions.^[163]^ The similar *J* versus *T* plots observed for the ITO/[Fe(H_2_B(pz)_2_)_2_(phen)](30 nm)/Al and ^TS^Au/[Fe(H_2_B(pz)_2_)_2_(phen)] (16.7 nm)/EGaIn junctions indicate higher conductance of the HS [Fe(H_2_B(pz)_2_)_2_(phen)] films produced by temperature‐induced SCO. A similar increase in conductivity upon LS→HS switching was observed for ^TS^Au/[Fe(HB(trz)_3_)_2_] (6.7 nm)/EGaIn junction.^[164]^ The situation may be different for the HS films produced by light irradiation at low temperatures, as established for the ITO/[Fe(H_2_B(pz)_2_)_2_(phen)]/Al optoelectronic junctions.

**Figure 14:**
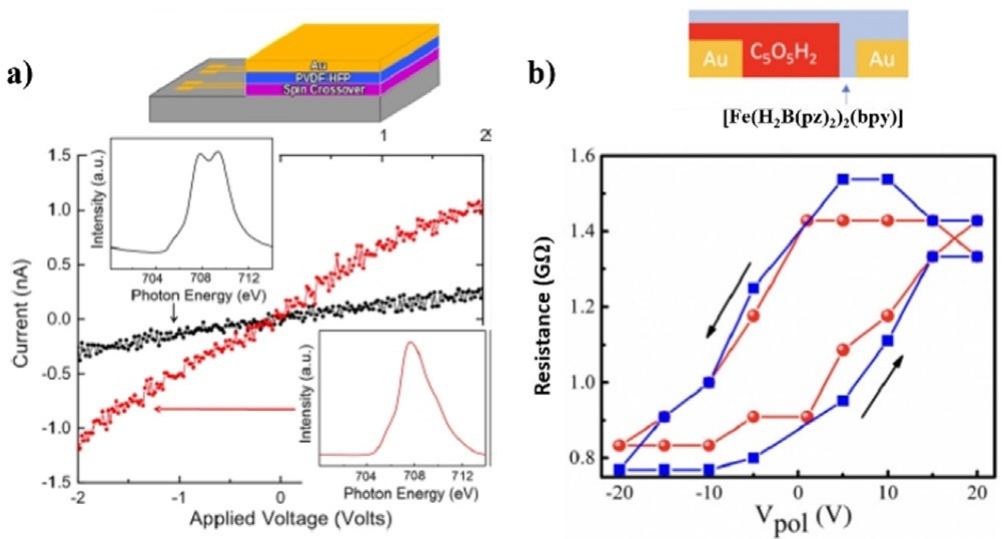
Conductance switching in [Fe(H_2_B(pz)_2_)_2_(bpy)] thin films on PVDF‐HFP and croconic acid ferroelectric substrates. a) The conductance traces originate from LS and HS states of the [Fe(H_2_B(pz)_2_)_2_(bpy)] film on PVDF‐HFP and are depicted as black and red traces, respectively; the device structure is shown above the current versus voltage plot.b) Plots of resistance versus poling voltage (*V*_pol_) for [Fe(H_2_B(pz)_2_)_2_(bpy)] on croconic acid. Reproduced with permission from ref. [121]. Copyright (2019) American Institute of Physics.

A voltage‐controlled isothermal nonvolatile resistance change in [Fe(H_2_B(pz)_2_)_2_(bpy)] films deposited on PVDF‐HFP and croconic acid ferroelectric substrates was demonstrated as depicted in Figure 14. As discussed in Section 3.2 (Figure 9), the spin‐state of [Fe(H_2_B(pz)_2_)_2_(bpy)] is pinned in either LS‐ or HS‐state depending on the direction of the ferroelectric polarization. The HS [Fe(H_2_B(pz)_2_)_2_(bpy)] film on PVDF‐PVP is more conductive than the LS film (Figure 14 a), and the conductance states of the film are retained even in the absence of applied voltage, evidencing the nonvolatile nature of the switching process. The nonvolatile spin‐state‐dependant conductance switching process between two resistance states is also elucidated in a [Fe(H_2_B(pz)_2_)_2_(bpy)] film on croconic acid as depicted in Figure 14 b; the resistance switching followed the ferroelectric switching of the croconic acid, conferring the film with bistable conductance switching characteristics.^[121]^

**Figure 15:**
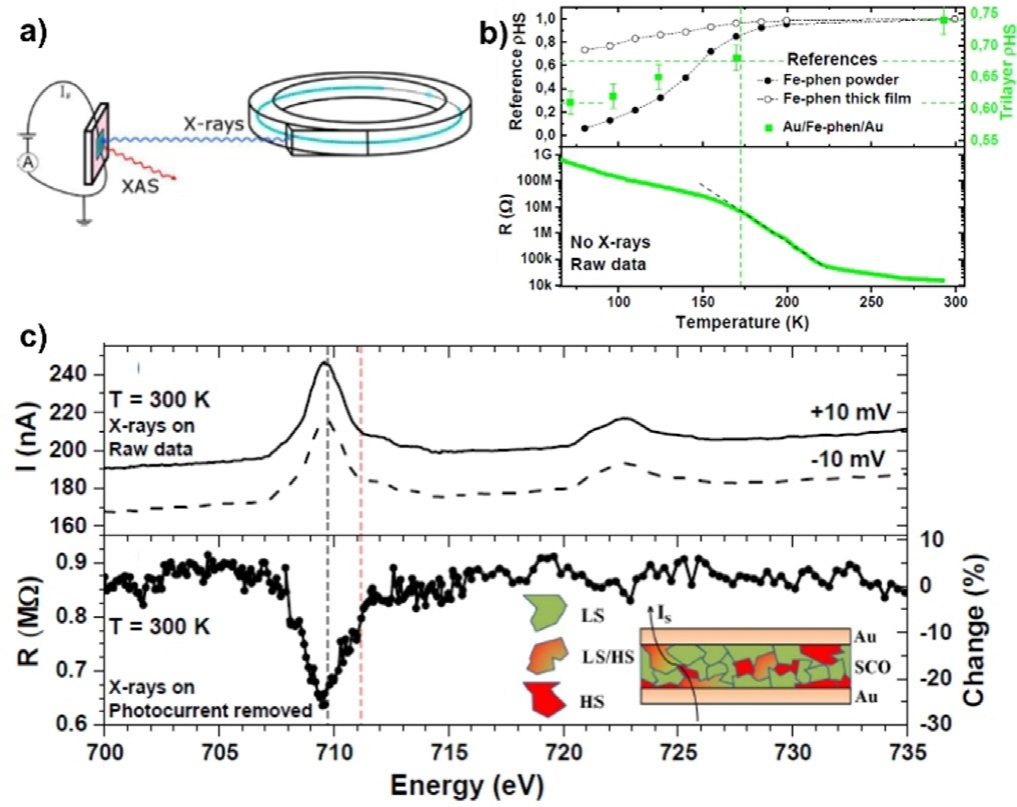
a) Schematic representation of simultaneous (device‐centric in operando) measurement of XAS and device resistance; b) temperature‐dependent (top) HS fraction of the bulk complex, 232 nm thick [Fe(H_2_B(pz)_2_)_2_(NH_2_‐phen)] film deposited on Au, and trilayer junction (actual device): Au/[Fe(H_2_B(pz)_2_)_2_(NH_2_‐phen)]/Au. The bottom panel shows the resistance evolution of the Au/[Fe(H_2_B(pz)_2_)_2_(NH_2_‐phen)]/Au junction in the absence of X‐ray irradiation; c) current (*I*) flowing across the Au/[Fe(H_2_B(pz)_2_)_2_(NH_2_‐phen)]/Au junction at ±10 mV under X‐ray irradiation at 300 K (top panel), and change in resistance at Fe L_3_ edge at 300 K indicating the HS mediated transport in the Au/[Fe(H_2_B(pz)_2_)_2_(NH_2_‐phen)]/Au junction, as depicted in the inset of the bottom panel. Reproduced with permission from ref. [104]. Copyright (2018) American Chemical Society.

The transport studies described so far have relied on separate measurements to link electronic transport and the spin‐state of the films. In a quest to capture the SCO‐mediated resistance variations during the device operation, device‐centric in operando XAS studies of Au/[Fe(H_2_B(pz)_2_)_2_(L^6^)] (42 nm)/Au junctions were carried out at 300 K while the device is in operation (Figure 15 a). Temperature‐dependent XAS studies revealed complete spin‐state switching of the bulk sample, whereas only 13 % of the molecules in the Au/[Fe(H_2_B(pz)_2_)_2_(L^6^)]/Au junction underwent SCO, as depicted in Figure 15 b. The corresponding temperature dependence of the device resistance showed discontinuities around the SCO region (*T*_1/2_) of the complex (green dotted line in Figure 15 b), indirectly establishing a link between the temperature‐dependent junction resistance and spin‐state of [Fe(H_2_B(pz)_2_)_2_(L^6^)].

The device‐centric in operando measurements performed at 300 K under an applied bias of ±10 mV showed the evolution of device current along the Fe L_3_ edge, indicating the involvement of Fe centers belonging to the HS [Fe(H_2_B(pz)_2_)_2_(L^6^)] complex in mediating transport (Figure 15 c). To unambigously support the involvement of the [Fe(H_2_B(pz)_2_)_2_(L^6^)] complex in mediating transport, the photocurrent originating due to the X‐ray irradiation of the device is substracted from the total resistance change and the resultant resistance is observed to be evolving along the Fe L_3_ edge, as shown in Figure 15 c (bottom panel). This supports the role of HS molecules in mediating transport. By comparing the material‐centric experiments (Figure 15 b) and in operando device‐centric measurements (Figure 15 c), a clear correlation between spin‐state and device resistance is elucidated.^[104]^

Temperature‐dependent *I–V* measurements of 200 nm thick films of [Fe(HB(pz)_3_)_2_] embedded between interdigitated gold electrodes revealed a gradual increase of the conductivity of the films in the first heating step until ≈350 K.^[124]^ The charge‐hopping‐mediated conductivity dropped above 350 K due to the onset of LS→HS switching: the LS‐state is more conductive than the HS‐state. Further heating of the sample, in the first heating step, above 370 K resulted in a drop of the conductivity due to the irreversible structural transformation from the metastable tetragonal to the stable and more insulating monoclinic form, which persisted in the subsequent *I–V* cycles. A read‐only memory (ROM) is fabricated exploiting the irreversible conductivity drop associated between the first and second cycles; information is written by heating the film above 370 K, that is, by inducing the irreversible tetragonal‐to‐triclinic structural transition, and the reading process is carried out by measuring the resistivity change of the sample at RT.^[92]^

In summary, the electrical conductance studies of the sublimable complexes revealed that the HS films of [Fe(phen)_2_(NCS)_2_], [Fe(H_2_B(pz)_2_)_2_(phen)], [Fe(H_2_B(pz)_2_)_2_(bpy)], [Fe(H_2_B(pz)_2_)_2_(NH_2_‐phen)], and [Fe(HB(trz)_3_)_2_] are more conductive than their LS counterparts. The smaller band gaps of the HS complexes, relative to the corresponding LS complexes, and closer energetic alignment of the HS FMOs with the Fermi levels of the electrodes rendered the HS films more conductive than the LS films. However, the more conductive nature of the LS films of [Fe(H_2_B(pz)_2_)_2_(phen)], and [Fe(HB(pz)_3_)_2_] studied in optoelectronic (5 K) and thermal junctions, respectively, cannot be explained based on FMO energies. A mechanistic pathway based on charge hopping, mediated by vibrational frequency differences associated with the LS→HS switching, is invoked to explain the conductivity modulations associated with [Fe(H_2_B(pz)_2_)_2_(phen)] films at 5 K.

### 4.3 SCO‐Based Microelectromechanical Systems

**Figure 16:**
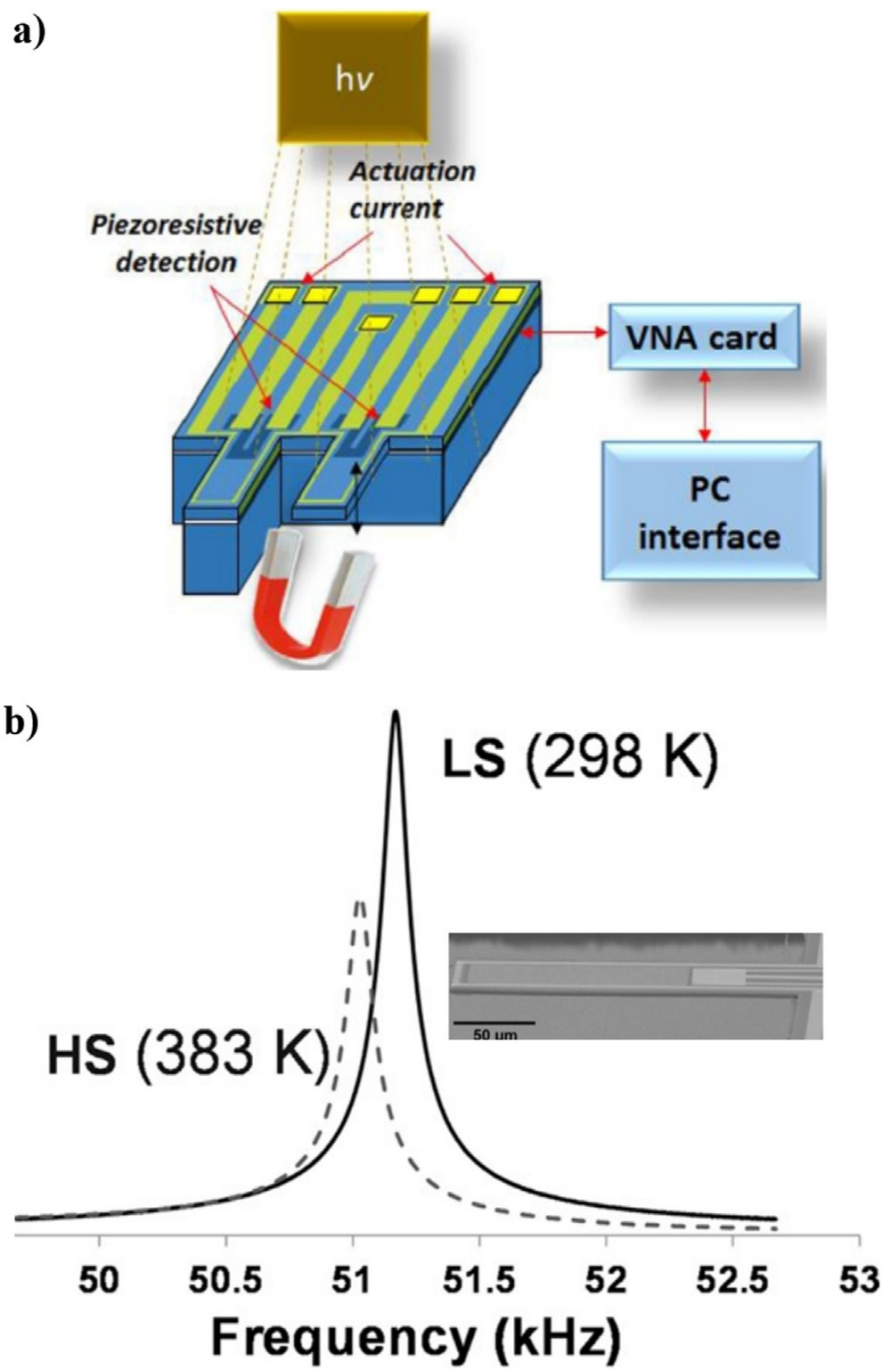
a) Schematic of the MEMS device comprising magnetic actuation and piezoresistive detection components. The device is magnetically actuated at its resonance frequency by passing current through a conducting path integrated onto the microcantilever. The detection of the mechanical vibrations is carried out by piezoresistors integrated at the cantilevers. b) Resonance curves of the microcantilever coated with 140 nm thick film of [Fe(HB(trz)_3_)_2_]: the LS→HS conversion caused about 66 Hz decrease in the resonance frequency of the cantilever. (a) is reproduced with permission from ref. [165]. Copyright (2016) American Institute of Physics.

A typical micro‐ or nanoelectromechanical system (MEMS or NEMS) device comprises electrical and mechanical components integrated into a silicon chip. The mechanical components such as microsensors respond to the external conditions, and the microelectronics components process the information received from the mechanical components and effect the desired change to the external environment. The sensitive nature of SCO complexes to external stimulus, such as light, temperature, or pressure, render them suitable to function as sensing and actuating elements in MEMS and NEMS devices. Moreover, the molecular nature of the SCO complexes is advantageous for the development of more efficient and smaller NEMS devices. A prototypical SCO‐based MEMS device is composed of an SCO thin film coated on a freestanding Si cantilever integrated with magnetic actuation and piezoresistive detection components (Figure 16 a). In response to the external stimulus, the film undergoes spin‐state switching, which effects a change in the unit cell parameters and the associated lattice‐volume expansion/compression. The elastic modulations taking place in the crystal lattice of an SCO complex couple with the underlying Si cantilever and the cantilever is actuated. A reversible change of the resonance frequency of the Si cantilever is also effected, when measured in dynamic mode, by such coupling, as depicted in Figure 16 b.^[11]^

A 200 nm film of [Fe(H_2_B(pz)_2_)_2_(phen)] coated on a Si cantilever (500 μm length, 120 μm width, and 20 μm thickness), which was integrated with a magnetic actuation–pizeoresistive detection system, acted as an actuator in a prototypical SCO‐based MEMS device. The mechanical coupling between the Si cantilever and the softening of the crystal lattice, brought in by the light‐induced LS→HS switching of the film at 10 K, is observed as a shift in the resonance frequency (Δ*f*_r_)=−0.52 Hz; that is, *f*_r_ decreased upon LS→HS switching.^[165]^ In a step towards realistic technological applications, a bistable MEMS operating under ambient conditions is demonstrated. The MEMS device is produced by coating a 140 nm thick film of [Fe(HB(trz)_3_)_2_] on a freestanding microcantilever (200 μm length, 50 μm width, and 2 μm thickness) integrated with a piezoresistive detection system. The temperature‐induced LS→HS switching of the film produced a reversible upward bending of the cantilever in the static mode. In the dynamic mode, that is, when the mechanical device was actuated at its resonance frequency, a resonance frequency (*f*_r_) decrease of about 66 Hz was measured as shown in Figure 16 b.^[166]^ Note the remarkable increase in the Δ*f_r_* observed for the smaller device based on [Fe(HB(trz)_3_)_2_] relative to the larger [Fe(H_2_B(pz)_2_)_2_(phen)]‐based device, indicating the utility of the sublimable SCO complexes to fabricate more efficient NEMS devices. An attempt to correlate the mechanical properties and the crystal structure of [Fe(HB(trz)_3_)_2_] revealed anisotropic variation in the unit cell parameters, causing an anisotropic variation of the elastic modulus of the complex upon LS→HS switching.^[167]^ A LS→HS switching mediated resonance frequency decrease is also observed in a MEMS device made of an organic microelectromechanical resonator coated with [Fe(H_2_B(pz)_2_)_2_(dmbpy)] (dmbpy=4,4′‐dimethyl‐2,2′‐bipyridine). The utility of the MEMS device as a light and temperature detector and as a nonvolatile memory was demonstrated.^[168]^

## 5 Summary and Outlook

A detailed account of advances made in the field of sublimable SCO complexes is presented. The SCO of the several hundred nm thick films of the complexes on a range of substrates resembled the bulk SCO behavior, indicating the dominant role of intermolecular interactions in determining the SCO characteristics in thick films. The increase in the cooperativity with increasing film thickness and bulk‐like SCO observed for [Fe(H_2_B(pz)_2_)_2_(phen)] on HOPG is a testimony to the role of intermolecular interactions in determining SCO parameters in thick films, and an indication of the weakening of interfacial interactions with increasing distance from the interfacial region. On the other hand, SCO of the sub‐monolayer and monolayer films of the complexes deposited on metallic substrates is blocked either due to fragmentation of the complexes or due to the interfacial constrains. Complete SCO at the sub‐monolayer scale is achieved for complexes deposited on passive HOPG; however, this result needs to be substantiated by studying a greater number of SCO complexes at sub‐monolayer coverage on HOPG. Despite much progress made in terms of achieving SCO at the molecular scale and in thick films, the realization of a sublimable SCO complex capable of undergoing abrupt and hysteretic SCO at or around RT both in the bulk and thin‐film states remains elusive: systematic investigations are necessary to obtain thin bistable films. Transport characteristics of the SCO complexes from the single‐molecule level to the thin‐film level evidenced higher conductance values associated with the HS complexes relative to their LS counterparts. The relatively low conductance of the HS [Fe(H_2_B(pz)_2_)_2_(phen)] in optoelectronic junctions at 10 K is a remarkable exception. The variation in conductivity upon spin‐state switching is utilized to fabricate memory architectures.

Despite much progress made and device architectures fabricated, areas that need more attention still remain. The chemical synthesis of designer functional sublimable SCO complexes is still in its infancy, and more systematic efforts are warranted in this direction. In this regard, it is desirable to design SCO complexes with an additional physical property, thus functional‐SCO complexes, which could be sublimed onto a suitable substrate with preservation of switching characteristics. Functional‐SCO complexes tailored with organic semiconductors,^[169]^ photoluminescent substituents,[^170^, ^171^, ^172^, ^173^] and self‐assembly‐inducing moieties[^105^, ^174^] are proposed. The high resistance of organic molecules is a bottleneck for the realization of active molecular electronic components for applications. The discovery of conducting polymers and developments made in the field of organic semiconductors led to molecular materials with applications in organic electronics and photovoltaics.[^175^, ^176^, ^177^, ^178^] Herein, we propose to study the spin‐state switching characteristics of sublimable complexes tethered with organic semiconductors both in the bulk and thin‐film states to obtain spin‐state‐dependent synergistic conductance modulation. By studying the spin‐state switching characteristics of luminescent SCO complexes, magneto‐optical correlations, that is, the synergistic coupling between luminescence and spin‐state, could be established, which may facilitate the optical addressing of SCO.[^170^, ^173^] On the other hand, appending SCO moieties with self‐assembly‐inducing substituents may facilitate the fabrication of spin‐state‐tunable nanoarchitectures via sublimation, which may enable the establishment of thickness and self‐assembly dependence of SCO at the nanoscale, as established for [Fe(H_2_B(pz)_2_)_2_(phen)] on HOPG.^[75]^ Chiral resolution of SCO complexes in the bulk and thin‐film states may result in novel magneto‐optical hybrids.

From the interfacial regime, the realization of spin‐state switchable films of molecular thickness/nanostructures on magnetically “active” ferromagnetic surfaces may facilitate the observation of bistable spinterface based on the presence (ON) or absence (OFF) of magnetic coupling between the HS or LS SCO center and the ferromagnetic substrate, respectively. Novel magneto‐electric hybrids taking advantage of the device‐suitable magnetic properties of SCO complexes and superior electrical and optical characteristics of the graphene could also be realized.[^151^, ^158^, ^179^] In this context, the self‐assembly of sublimable SCO complexes on a graphene surface could be explored; the observation of efficient spin‐state switching at the single‐molecule level on HOPG encourages further investigation in this direction. The possibility of doping graphene, analogous to the surface chemical doping of graphene,[^180^, ^181^] by an SCO complex may facilitate the reversible modulation of charge carriers, “switchable‐doping,” in graphene depending upon the spin‐state of an SCO complex, a prerequisite for practical realization of graphene‐based electronics. So far, the SCO of sublimable complexes has been studied mostly on metallic and HOPG substrates. A reasonable continuation in this direction would be probing the SCO of sublimable complexes on 2D materials, for example, MoS_2_ and CrI_3_, with intriguing magnetic, optical, and electrical characteristics.[^182^, ^183^, ^184^, ^185^, ^186^, ^187^, ^188^, ^189^]

Overall, a great deal of progress has been made in terms of studying on‐surface switching characteristics of sublimable SCO complexes and the fabrication of device architectures showing spin‐state‐dependent conductivity modulation. More systematic and relentless efforts may lead to the fundamental understanding of spin‐state switching process in the nanostructured environments along with the realization of futuristic molecular electronics and spintronics devices based on functional vacuum‐sublimable SCO complexes.

## Conflict of interest

The authors declare no conflict of interest.

## Biographical Information

*Kuppusamy Senthil Kumar obtained his PhD from IIT Madras under the guidance of Prof. Archita Patnaik. Currently, he is a postdoctoral researcher in the group of Prof. Mario Ruben at the Institute of Nanotechnology (INT), Karlsruhe Institute of Technology (Germany). He is interested in studying the spin‐crossover (SCO) phenomenon in the bulk and at the nanoscale as well as the development of novel quantum materials*.

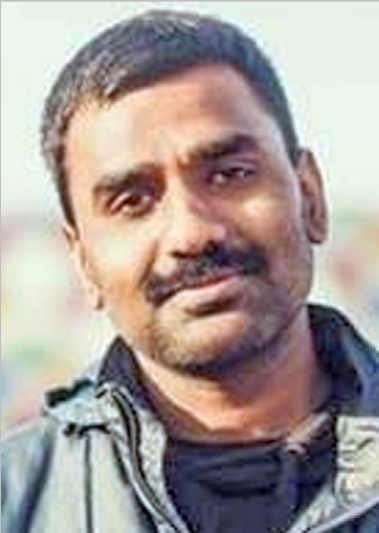

## Biographical Information

*Mario Ruben obtained his PhD in 1998 from the University of Jena (Germany). During a DAAD postdoctoral fellowship, he worked in Prof. J.‐M. Lehn's research group at the ISIS in Strasbourg. In 2001, he moved to the Institute of Nanotechnology in Karlsruhe (Germany) and in 2010, he accepted a position as Professor Conventionné at the Université de Strasbourg. In 2013, he became Full Professor for Molecular Materials at the Institutes of Inorganic Chemistry and of Nanotechnology at KIT. His research interests involve the design, synthesis, and characterization of functional molecules and their implementation in operational (quantum) devices*.

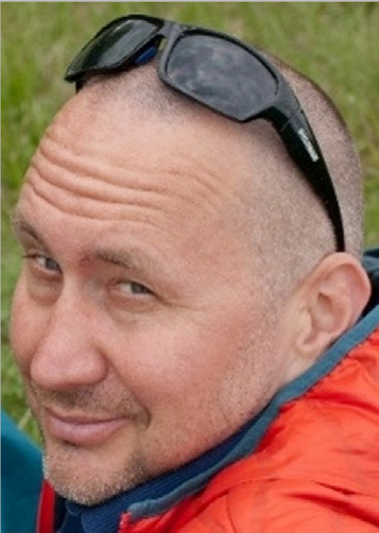

## Supporting information

As a service to our authors and readers, this journal provides supporting information supplied by the authors. Such materials are peer reviewed and may be re‐organized for online delivery, but are not copy‐edited or typeset. Technical support issues arising from supporting information (other than missing files) should be addressed to the authors.

Supplementary
