# Supplementary material for: Sublimable Spin‐Crossover Complexes: From Spin‐State Switching to Molecular Devices
Source: Angew Chem Int Ed Engl. 2020 Oct 29;60(14):7502–21. doi: 10.1002/anie.201911256 (PMC8048919; doi:10.1002/anie.201911256)
Supplement: Supplementary file 1 — Supplementary [file ANIE-60-7502-s001.pdf]

Angewandte Chemie
Eine Zeitschrift der Gesellschaft Deutscher Chemiker

Supporting Information

Sublimable Spin-Crossover Complexes: From Spin-State Switching to Molecular Devices

Kuppusamy Senthil Kumar and Mario Ruben**

anie_201911256_sm_miscellaneous_information.pdf

Supplimentary Information

Sublimable Spin-crossover Complexes: From Spin-State Switching to Molecular Devices

Kuppusamy Senthil Kumar,*[a] and Mario Ruben*[a, b]

Content: ***$\chi_M T$ versus T* plot of sublimable complexes in the bulk state**

Figure S1 The bulk state SCO behavior of [Fe(dpepd)(NCS)$_2$]

Figure S2 The bulk state SCO behavior of [Fe(HB(pz)$_3$)$_2$] and [Fe(HB(3,5-(CH$_3$)$_2$(pz)$_3$)$_2$]

Figure S3 The bulk state SCO behavior of [Fe(qnal)$_2$]·CH$_2$Cl$_2$ and [Fe(qnal)$_2$]

Figure S4 The bulk state SCO behavior of [Fe(pypyr(CF$_3$)$_2$)$_2$(phen)]

Figure S5 The bulk state SCO behavior of [Fe(pap)$_2$]ClO$_4$·H$_2$O

References

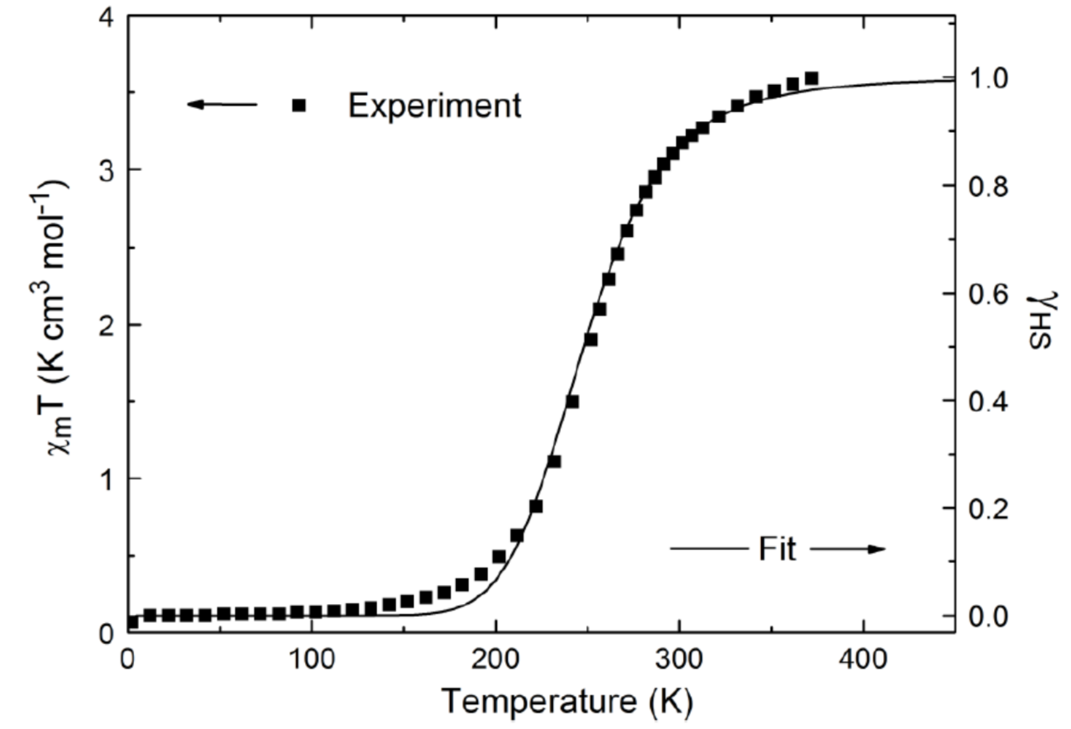

Figure S1. The bulk state SCO behavior ($\chi_M T$ *versus* T plot) of [Fe(dpepd)$(NCS)_2$]. Reproduced with permission from reference 1, copyright (2012) American Chemical Society.[1]

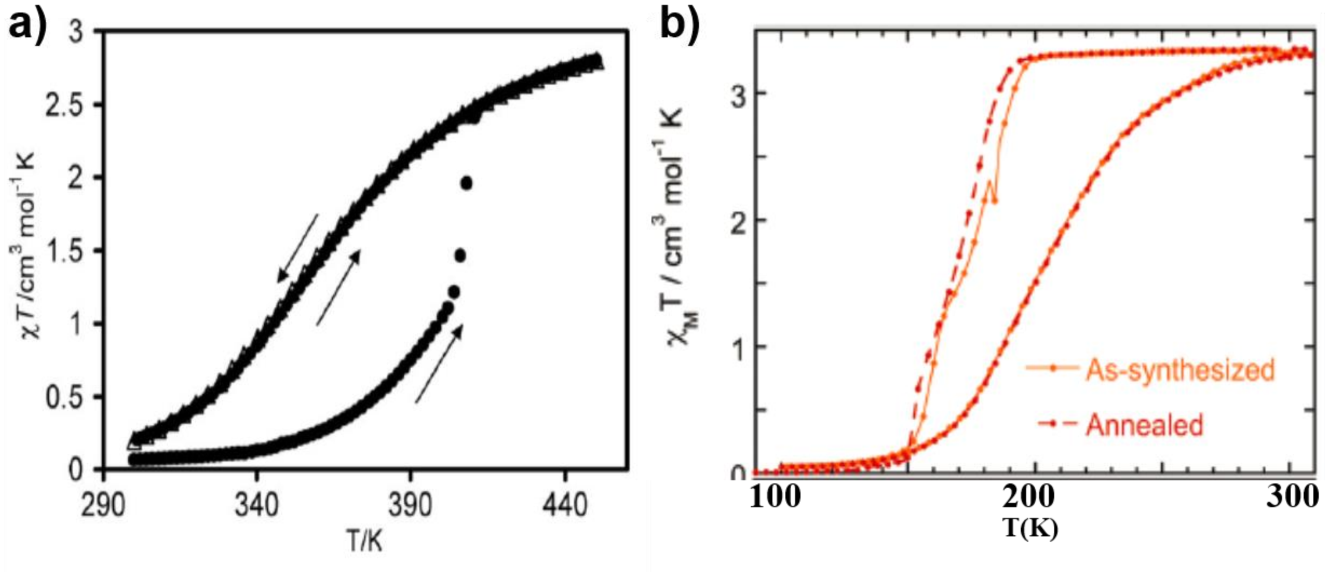

Figure S2. The bulk state SCO behavior ($\chi_M T$ *versus* T plots) of (a) $[Fe(HB(pz)_3)_2]$; reproduced with permission from reference 2, copyright (2009) Royal Society of Chemistry[2] and (b). $[Fe(HB(3,5\text{-}(CH_3)_2(pz)_3)_2]$; reproduced with permission from reference 3, copyright (2017) Royal Society of Chemistry.[3]

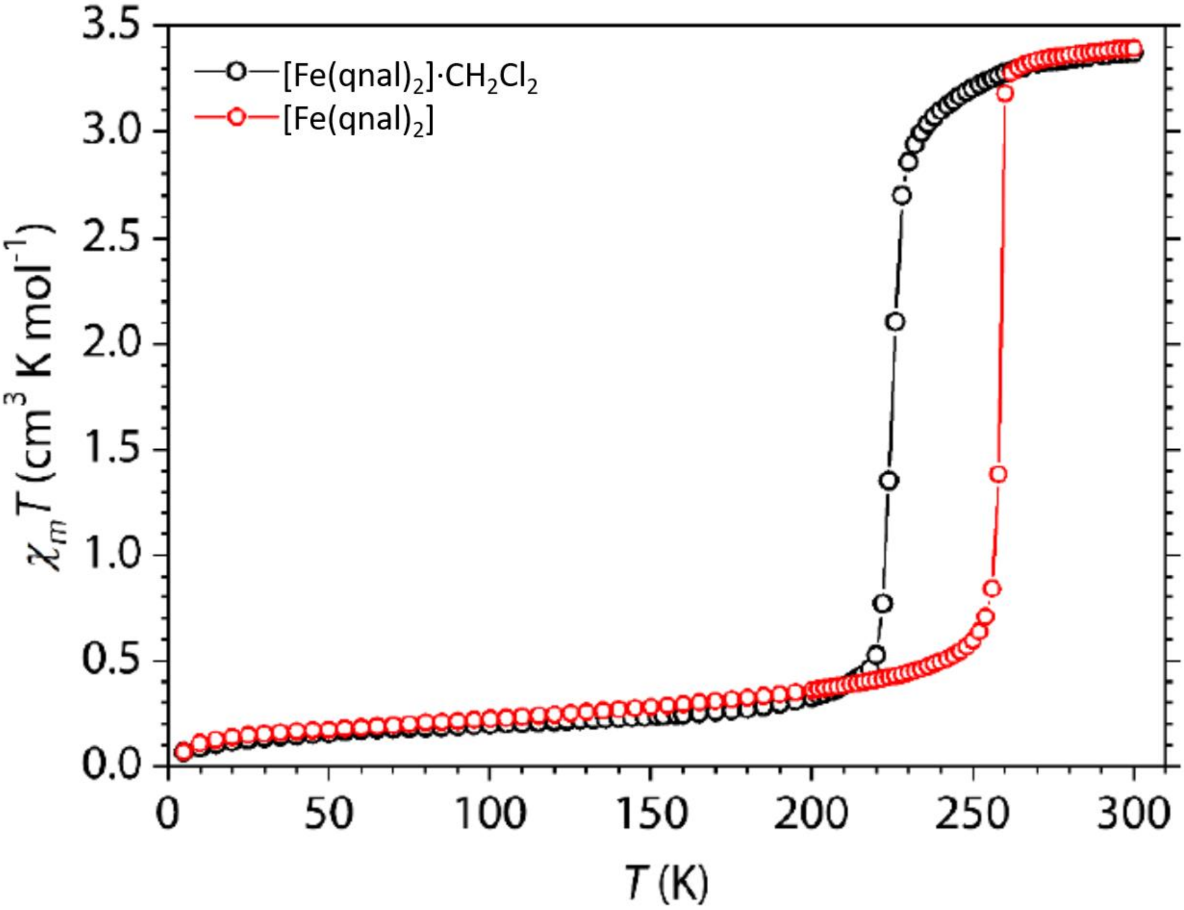

Figure S3. The bulk state SCO behavior ($\chi_M T$ *versus T* plot) of $[Fe(qnal)_2]\cdot CH_2Cl_2$ (Black traces) and $[Fe(qnal)_2]$ (Red traces). Reproduced with permission from reference 4, copyright (2018) Royal Society of Chemistry.[4]

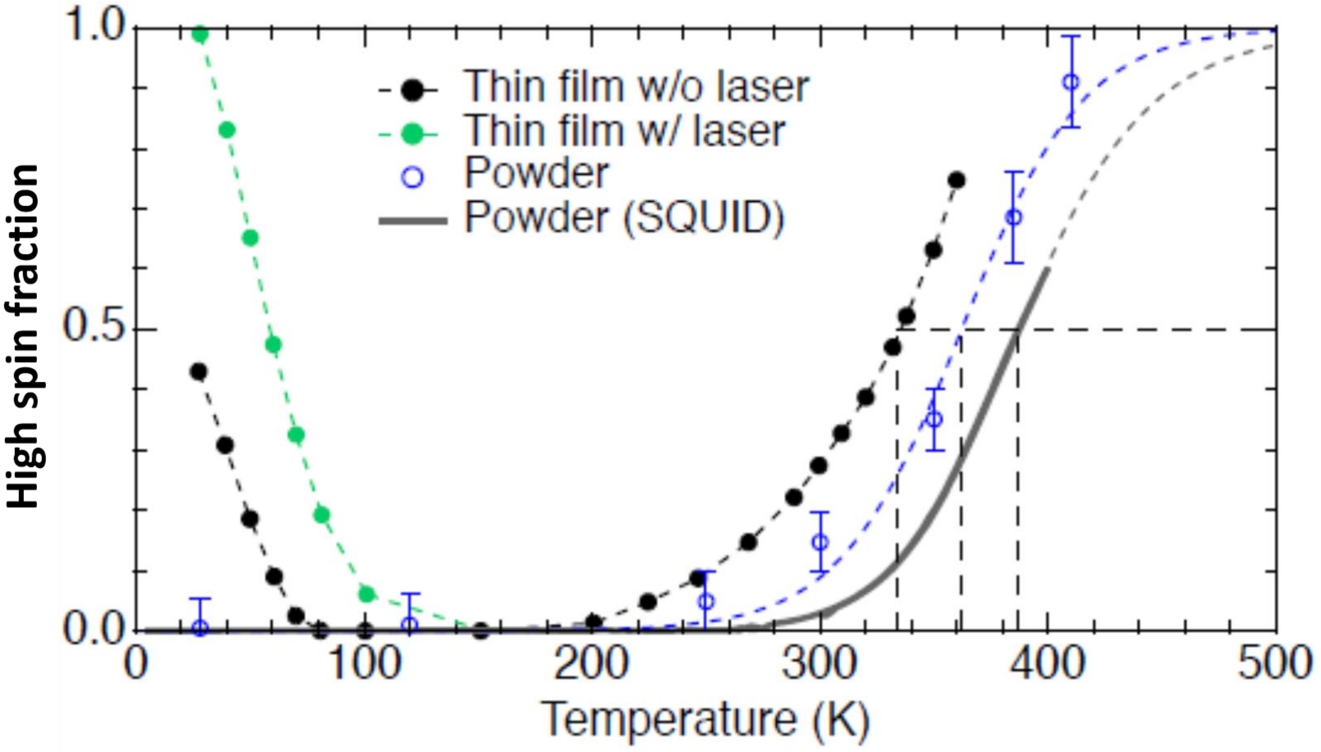

Figure S4. The bulk state SCO behavior ($\chi_M T$ *versus T* plot) of [Fe(pypyr(CF$_3$)$_2$)$_2$(phen)] designated as Powder (SQUID) in the picture. Reproduced with permission from reference 5, copyright (2018) American Chemical Society.[5]

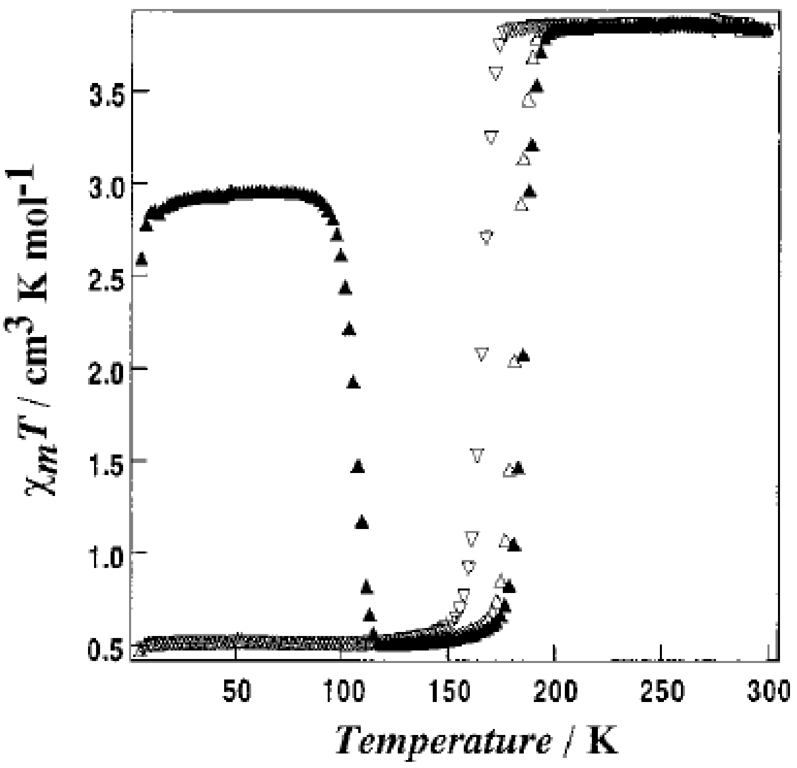

Figure S5. The bulk state SCO behavior ($\chi_M T$ *versus T* plots) of $[Fe(pap)_2]ClO_4{\cdot}H_2O$. Reproduced with permission from reference 6, copyright (2000) American Chemical Society.[6]

References

[1] M. Bernien, D. Wiedemann, C. F. Hermanns, A. Krüger, D. Rolf, W. Kroener, P. Müller, A. Grohmann, W. Kuch, *J. Phys. Chem. Lett.* **2012**, *3*, 3431–3434.

[2] L. Salmon, G. Molnár, S. Cobo, P. Oulié, M. Etienne, T. Mahfoud, P. Demont, A. Eguchi, H. Watanabe, K. Tanaka, et al., *New J. Chem.* **2009**, *33*, 1283.

[3] O. Iasco, M.-L. Boillot, A. Bellec, R. Guillot, E. Rivière, S. Mazerat, S. Nowak, D. Morineau, A. Brosseau, F. Miserque, et al., *J. Mater. Chem. C* **2017**, *5*, 11067–11075.

[4] M. Atzori, L. Poggini, L. Squillantini, B. Cortigiani, M. Gonidec, P. Bencok, R. Sessoli, M. Mannini, *J. Mater. Chem. C* **2018**, *6*, 8885–8889.

[5] S. Rohlf, M. Gruber, B. M. Flöser, J. Grunwald, S. Jarausch, F. Diekmann, M. Kalläne, T. Jasper-Toennies, A. Buchholz, W. Plass, et al., *J. Phys. Chem. Lett.* **2018**, *9*, 1491–1496.

[6] S. Hayami, Z. Gu, M. Shiro, Y. Einaga, A. Fujishima, O. Sato, *J. Am. Chem. Soc.* **2000**, *122*, 7126–7127.
